# Insights into Molecular Mechanisms of Polyphenolic Compounds from *Helichrysum italicum* by Inverse Molecular Docking Fingerprint Approach

**DOI:** 10.3390/ph19040647

**Published:** 2026-04-21

**Authors:** Veronika Furlan, Vid Ravnik, Urban Bren, Marko Jukić

**Affiliations:** 1 Faculty of Chemistry and Chemical Engineering, University of Maribor, Smetanova 17, SI-2000 Maribor, Slovenia; veronika.furlan@um.si (V.F.); vid.ravnik2@um.si (V.R.); 2 Faculty of Mathematics, Natural Sciences and Information Technologies, University of Primorska, Glagoljaška 8, SI-6000 Koper, Slovenia; 3 Institute of Environmental Protection and Sensors, Beloruska Ulica 7, SI-2000 Maribor, Slovenia

**Keywords:** *Helichrysum italicum*, polyphenolic compounds, protein targets, inverse molecular docking fingerprints, molecular mechanisms, synergistic effects, mode of action, biological effects

## Abstract

**Background/Objectives**: Natural compounds occupy a pharmacologically rich chemical space, characterized by abundant scaffolds, extensive functional group elaboration, and defined stereochemistry. In this context, *Helichrysum italicum*, a Mediterranean medicinal plant, represents a valuable source of polyphenols with multiple biological and pharmacological activities. **Methods**: Here, we introduce an inverse molecular docking fingerprint approach to systematically investigate eight major *Helichrysum italicum* polyphenols, including α-pyrones (arzanol, ethylpyrone), flavonols (gnaphaliin, kaempferol, quercetin), and flavanones (naringenin, pinocembrin, hesperetin). More than 40,000 human protein structures from the Protein Data Bank were screened to generate target-based inverse docking score fingerprints for each compound. **Results**: Hierarchical clustering of these fingerprints revealed shared binding patterns among structurally related polyphenols and enabled hypothesis generation regarding potential synergistic effects. Notably, favorable interactions were identified with PPARG and CARM1, supporting therapeutic relevance in inflammation and cancer, alongside additional targets associated with neurodegeneration and bone metabolism. **Conclusions**: This study establishes inverse docking fingerprints as a robust, mechanism-oriented method for natural product research and highlights *Helichrysum italicum* polyphenols as starting points for medicinal chemistry and drug discovery.

## 1. Introduction

In recent years, medicinal plants with health-promoting properties have gained considerable agricultural significance [1]. *Helichrysum italicum*, also known as immortelle or everlasting, represents an important medicinal plant with numerous beneficial health effects. Interest in immortelle originates from its applications in the traditional medicine of Mediterranean countries for the treatment of inflammatory and allergic conditions, such as asthma and skin inflammation [2]. *Helichrysum italicum* represents an aromatic shrub, 30 to 70 cm tall, with small yellow flowers that emit a strong, persistent curry-like scent [3]. It grows mainly on dry, sandy–stony soils in Mediterranean regions, from sea level up to an altitude of 2200 m [4]. It is adapted to arid conditions with limited access to water [5] and usually flowers from June (in some places as early as May) to August or September [6]. It is named the immortal flower, as its flowers do not wither and retain their vibrant yellow color even after being picked and dried [7]. As a typical xerophytic plant, it thrives mainly in sandy and rocky areas, often on carbonate and skeletal soils [6]. Cultivation of *Helichrysum italicum* has become increasingly prevalent in several European regions, including Corsica, Italy, Hungary, Bosnia and Herzegovina, and Croatia. Comparative analyses of *Helichrysum italicum* samples from different regions of the Mediterranean basin have shown significant variability in the composition of bioactive compounds [8]. Various factors, such as the ecological and climatic conditions of the cultivation sites, geographical location, soil characteristics (including texture and pH), the developmental stage of the plant, plant subspecies, drying method, storage, and the selected extraction process [9] can significantly affect the quality and composition of its plant extract and essential oils. Due to their remarkable antioxidative properties, immortelle extracts and essential oils excel in anti-wrinkle and anti-aging applications [10]. Known therapeutic applications also include wound healing, treatment of gallbladder and bladder diseases, and analgesic use [8]. Imortelle is also applied for the treatment of various skin, respiratory, and digestive inflammations, as well as for the elimination of varicose veins. In addition to their health benefits, immortelle flowers are also used as spices, in food flavoring, and as natural preservatives in foods [10].

**Figure 1:**
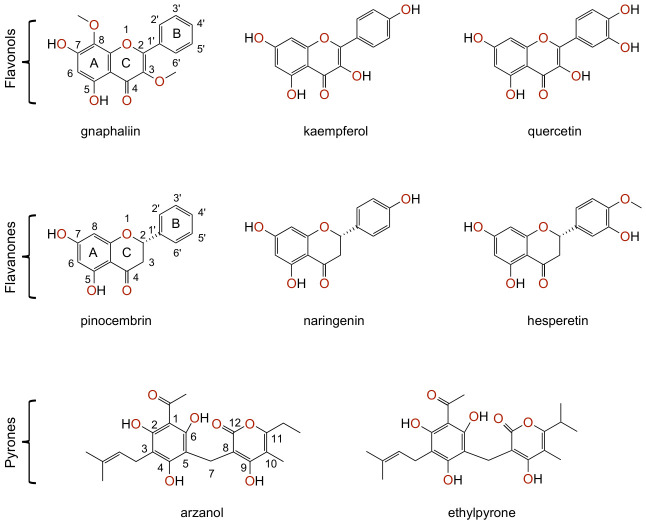
Structures of major *Helichrysum italicum* polyphenolic compounds from flavonol, flavanone and α-pyrone classes.

*Helichrysum italicum* extracts and essential oils form a rich source of a wide variety of chemical classes, namely, polyphenolic compounds, acetophenones, tremetones, monoterpenes, sesquiterpenes, and triterpenes [2]. The extracts are especially rich in polyphenolic compounds, namely, flavonoids, and α-pyrones, while the essential oils contain mostly monoterpenes and sesquiterpenes [2]. *Helichrysum italicum* extracts with a high content of non-volatile polyphenolic compounds were reported to exhibit antioxidative, antiinflammatory [10], antibacterial [11], antifungal [12], and antiviral [13] effects. Phenolic and polyphenolic compounds represent a diverse class of over 10,000 known compounds characterized by the presence of one or more aromatic rings with one or more hydroxyl groups attached. As secondary plant metabolites, they play a crucial role in defense mechanisms against oxidative stress, UV radiation, pathogens, and parasitic organisms [14]. Polyphenolic compounds demonstrated a protective effect on healthy cells, while they exert cytotoxic effects on malignant cells [15]. The quality of the extracts is primarily determined by the concentration of specific flavonoids such as gnaphaliin, kaempferol, quercetin, pinocembrin, and naringenin as well as the prenylated α-pyrone–phloroglucinol heterodimer arzanol (Figure 1).

Structurally, flavonoids contain a 15-carbon (C6-C3-C6) skeleton comprising two benzene rings (A and B) linked by a heterocyclic C ring, forming the phenylchromen-4-one scaffold [16]. Differences in oxygenation patterns, hydroxylation, methoxylation, and C2–C3 saturation generate distinct flavonoid subclasses [17], and these structural modifications are directly linked to their biological effects. For example, the presence of a C2=C3 double bond conjugated with the 4-oxo group and hydroxylation at C3 increases planarity and electron delocalization, which enhances antioxidant and anti-inflammatory activity, as demonstrated for quercetin and kaempferol [18]. Conversely, saturation of the C2-C3 bond, characteristic of flavanones, reduces planarity and alters how these compounds interact with target proteins [19]. Furthermore, hydroxylation patterns on the B-ring strongly modulate radical scavenging activity, where an ortho dihydroxy (catechol) configuration markedly increases antioxidant capacity, consistent with observations for quercetin compared to kaempferol [17]. Major *Helichrysum italicum* flavonoids illustrate these relationships. Gnaphaliin, the predominant flavonol [18], contains hydroxyl groups at C5 and C7 and methoxy substituents at C3 and C8, a pattern that increases hydrophobicity and may facilitate interactions with non-polar protein pockets. Kaempferol [20] contains four hydroxyl groups (C3, C5, C7, C4′) and therefore exhibits strong hydrogen bonding and radical scavenging abilities, while quercetin possesses an additional hydroxyl group at C5′, further enhancing its antioxidant potency through improved electron donation and stabilization of phenoxyl radicals [18]. The biological activities of flavanones from *H. italicum* differ from those of flavonols due to the absence of the C2=C3 double bond, which increases their conformational flexibility. Naringenin, with hydroxyl groups at C5, C7, and C4′ [19], can form multiple hydrogen bonds relevant to modulation of inflammatory pathways. Pinocembrin [21], lacking the C4′ hydroxyl group, is more hydrophobic and has been reported to have higher membrane permeability and bioavailability. Hesperetin contains a methoxy substituent at C4′, which further increases lipophilicity and affects the compound’s interactions with both proteins and membrane environments [19]. Both flavonols and flavanones occur in *Helichrysum italicum* predominantly in glycosylated forms, with tiliroside (kaempferol-3-O-(6-O-trans-p-coumaroyl-β-glucopyranoside)) being one of the major constituents [2]. Quantitative LC-MS studies consistently reported that flavonoid glycosides are more abundant than their aglycones, reflecting the typical biosynthetic storage form of polyphenols in plant tissues [18]. Glycosylation enhances solubility, stability, and intracellular transport of polyphenols, because sugar conjugation increases hydrophilicity and protects phenolic hydroxyl groups from oxidation and degradation [17]. However, in biological systems, these glycosides are typically hydrolyzed by intestinal β-glucosidases or by microbial enzymes, releasing the aglycone, which is the form generally responsible for binding to protein targets and exerting biological effects. Aglycones display higher affinity toward enzymes, receptors, and transcription factors due to their increased lipophilicity and ability to form stronger intermolecular interactions with amino acid residues. Therefore, polyphenol glycosides predominate in the plants and play key roles in solubility and transport, while the aglycones after hydrolysis are typically the primary bioactive species interacting with molecular targets in vivo [22].

Flavonols kaempferol and quercetin demonstrated anticarcinogenic effects by the inhibition of tumor cell proliferation, induction of apoptosis, and modulation of signaling pathways such as PI3K/Akt and NF-κB, leading to the inhibition of angiogenesis and metastasis [23]. Similarly, flavanones naringenin and hesperetin induced anticarcinogenic effects through suppressing oxidative stress and inflammatory mediators associated with carcinogenesis. Moreover, flavonols and flavanones downregulate pro-inflammatory cytokines (IL-6, TNF-α) and inhibit NF-κB activation as well as exhibit neuroprotective effects [23]. Quercetin and kaempferol also demonstrated antibacterial and antiviral effects by disrupting microbial membranes and inhibiting nucleic acid synthesis [24], while naringenin and pinocembrin exhibited strong antibacterial activity through synergistic effects with antibiotics [25].

Arzanol, a naturally occurring heterodimeric acylphloroglucinol α-pyrone, represents one of the major compounds from *Helichrysum italicum* [26], which is reported to exhibit antiinflammatory, antioxidative [10], antimicrobial [11], and antiviral effects [13]. Acylphloroglucinols are derivatives of the compound known as phloroglucinol (benzene-1,3,5-triol), which are composed of two or more rings bound together through methylene bridges. Structurally, arzanol contains a heterocyclic α-pyrone moiety connected through a methylene bridge to the phloroglucinol moiety [27]. Arzanol exhibits four hydroxyl groups at positions C2, C4, C6, and C9 as well as two keto groups at C1 and C12. Structurally similar ethylpyrone, which is also found in *Helichrysum italicum* extracts, contains an additional methyl group at position C13 [18]. The structures of arzanol and ethylpyrone differ from the flavonoids as they lack the flavonoid-specific C6-C3-C6 backbone; however, they contain a similar number of hydroxyl groups. Arzanol demonstrated antioxidative activity by reducing lipid peroxidation in vitro, and by reducing tert-butyl hydroperoxide (TBH)-induced oxidative stress in VERO cells [28]. Arzanol also inhibited HIV-1 replication in T cells (IC_50_ ≈ 5 μM), prevented activation of nuclear factor kappa beta (NF-κB) and pro-inflammatory mediators, namely, interleukins (IL)-1β, IL-6, IL-8, prostaglandin E2 (PGE2), and tumor necrosis factor alpha (TNF-α) in lipopolysaccharide (LPS)-induced monocytes [3]. Moreover, arzanol also demonstrated the inhibition of inducible microsomal PGE2 synthase (mPGES)-1, cyclooxygenase 1 (COX-1), and 5-lipoxygenase (5-LO) in vitro with IC_50_ values ranging from 0.4 to 9 μM, and significantly reduced inflammatory response in the carrageenan-induced pleurisy in rats as well as decreased the PGE2 levels in the pleural exudates [29].

Polyphenolic compounds can interact with a multitude of different protein targets, which leads to a challenging evaluation of their biological activities in in vitro and in vivo studies. It is also important to emphasize that *Helichrysum italicum* extracts represent complex mixtures of different compounds that can act synergistically while minimizing the potential adverse side effects of individual compounds. This complexity makes chemical analyses even more demanding and makes it difficult to determine which compound or combination of compounds is responsible for the specific biological effect. Consequently, it is important to study the molecular mechanisms of individual polyphenolic compounds in order to understand their pharmacological and synergistic effects, which could increase their potential in medical applications. Previous in silico studies have already explored binding mechanisms of arzanol [30], gnaphaliin [31], kaempferol [32], quercetin [33], pinocembrin [34], hesperetin [35], and naringenin [36] to specific protein targets; however, the studies focusing on the identification of new potential protein targets of *Helichrysum italicum* polyphenols and their binding patterns are still lacking in the scientific literature.

Inverse molecular docking represents a computational approach in which a promiscuous ligand is docked into the binding sites of a database of protein structures (ProBiS-Dock database [37] in this study). This approach reverses the traditional high-throughput virtual screening procedure, where a large virtual library of small ligands is docked into a specific protein target. In our previous studies, inverse molecular docking has been successfully applied to identify potential new protein targets and modes of action of natural polyphenolic compounds [38,39]. It was also successfully used in drug repurposing and to predict molecular mechanisms behind the adverse side effects of synthetic drugs [40]. Moreover, the inverse molecular docking fingerprint methodology has been proven successful in the identification of similar effects of major and minor cannabinoids on human protein targets [41]. To the best of our knowledge, the inverse molecular docking fingerprint approach has not yet been utilized on a set of major polyphenols from *Helichrysum italicum* extracts against all human protein targets. The aim of the present study is, therefore, to apply the in-house developed inverse molecular docking fingerprint method based on hierarchical clustering on a set of eight major polyphenolic compounds from *Helichrysum italicum* extracts in order to analyze the similarities and differences in their binding patterns. With the inverse molecular docking fingerprint approach, it is also possible to support future mode of action (MOA) studies of polyphenolic compounds and to suggest their mechanisms of action, which could lead to the development of potential medicinal applications of *Helichrysum italicum* extracts or their individual polyphenols.

## 2. Results and Discussion

### 2.1. Hierarchical Clustering of Inverse Molecular Docking Fingerprints

**Figure 2:**
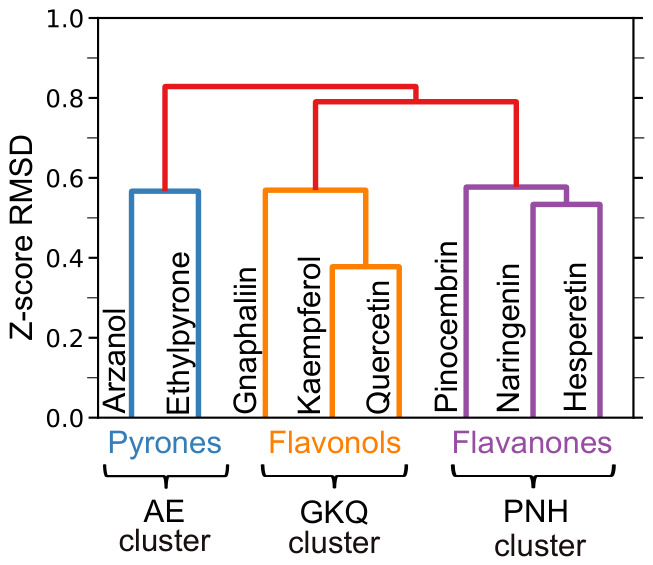
Hierarchical clustering of ligand inverse molecular docking fingerprints dendrogram using Ward linkage, derived from the complete pairwise Z-score RMSD matrix of investigated polyphenol docking into human protein targets. Abbreviations: AE: arzanol and ethylpyrone cluster, GKQ: gnaphaliin, kaempferol, and quercetin cluster, PNH: pinocembrin, naringenin, and hesperetin cluster.

Agglomerative clustering with Ward linkage was applied to analyze “all-against-all” fingerprint RMSD matrix, R. From the resulting dendrogram, presented in (Figure 2), three distinct clusters of investigated *Helichrysum italicum* polyphenols can be observed (separated by RMSD>0.75).

The first major cluster contains flavanone-class polyphenols pinocembrin, naringenin, and hesperetin (PNH), while flavonols gnaphaliin, kaempferol, and quercetin (GKQ) form the second major cluster. The third cluster consists of α-pyrone representatives arzanol and ethylpyrone. The clusters will be referred to by their respective polyphenol classes, namely, the flavanone, flavonol, and α-pyrone clusters, as the inverse molecular docking fingerprint clustering reproduces the classification of the polyphenols based on their chemical structure (Figure 1). According to the scientific literature, the flavonols quercetin and kaempferol demonstrated affinity to several common protein targets, namely, tumor protein p53 (TP53) [42], tumor necrosis factor (TNF), matrix metalloproteinase-2 (MMP2), signal transducer and activator of transcription 3 (STAT3), mitogen-activated protein kinase family (MAPK1/MAPK3) [43], and peroxisome proliferator-activated receptor alpha (PPAR-α) [44]. Kaempferol and quercetin also demonstrated a similar binding mode and docking score value (≈24.17 kcal/mol and ≈25.17 kcal/mol, respectively) to peroxisome proliferator-activated receptor gamma in a computational study, combining molecular docking and molecular dynamics simulations [45]. On the other hand, gnaphaliin represents the most underexplored flavonol from *Helichrysum italicum* with limited studies on protein targets like phosphodiesterases, specifically phosphodiesterase type 5 (PDE5) [46]. In the scientific literature, it was also reported that the flavanones naringenin, pinocembrin, and hesperetin demonstrated affinity to similar protein targets, namely, nuclear factor erythroid 2-related factor 2 (Nrf2), heme oxygenase-1 (HO-1) [47], RAC-alpha serine/threonine-protein kinase (Akt), mitogen-activated protein kinases (MAPK1, MAPK3, and MAPK8) [48,49], and nuclear factor kappa-light-chain-enhancer of activated B cells (NF-κB) [47,49], which is indeed in agreement with our clustering results. α-Pyrone-class polyphenols arzanol and ethylpyrone represent important natural compounds from *Helichrysum italicum*. For arzanol, the scientific literature reports human dihydroorotate dehydrogenase (hDHODH) [50], brain glycogen phosphorylase (bGP) [51], NF-κB [29], sirtuin 1 [52], microsomal prostaglandin E synthase-1, and 5-lipoxygenase [29] as confirmed protein targets, while, to the best of our knowledge, there are no previously reported protein targets of ethylpyrone.

Within the flavonol cluster, kaempferol and quercetin are separated from gnaphaliin by a RMSD≈0.58, while the differences in the flavanone cluster between naringenin and hesperetin with pinocembrin are less pronounced, with the same difference (RMSD≈0.58). Interestingly, the difference between flavonol and flavanone clusters was only slightly lower (RMSD≈0.79) than the difference between the α-pyrone cluster and both flavonoid clusters (RMSD≈0.81). The results of our study for the first time demonstrated that the inverse molecular docking fingerprints of structurally related α-pyrone-class polyphenols arzanol and ethylpyrone are indeed similar. Arzanol and ethylpyrone represent particularly promising compounds for further studies, as the identified protein binding patterns provide valuable insights into their potential beneficial pharmacological effects, which remain underexplored in the scientific literature.

It can be hypothesized that the rigidity of α-pyrone, flavonol, and flavanone scaffolds, the number of hydroxyl groups, and the alkyl side-chain lengths significantly contribute to the observed clustering. Flavonols kaempferol and quercetin differ only in the presence of one hydroxyl group at the position C5′ (RMSD≈0.38), while gnaphaliin lacks hydroxyl groups at the positions C3, C4′, and C5′, resulting in a higher RMSD of ≈0.58 within the flavonol cluster. The same trend can also be observed in the flavanone cluster, where naringenin and hesperetin differ only in the hydroxyl group at the position C4′ or C5′ (RMSD≈0.56), while pinocembrin demonstrated a slightly higher difference due to the absence of hydroxyl groups at the positions C4′ and C5′ (RMSD≈0.58). The difference in binding patterns between arzanol and ethylpyrone can be attributed to the change in the alkyl side-chain length at the position C11, resulting in RMSD≈0.58. Further analysis of the inverse molecular docking fingerprint RMSD shows that compounds with the most similar fingerprints differ only in the number and positions of hydroxyl groups or in the case of the α-pyrone cluster in the alkyl side-chain length. Interestingly, the differences between arzanol and ethylpyrone from the α-pyrone cluster are similar to the differences between polyphenols from flavonol and flavanone clusters (RMSD≈0.58). This indicates that the change in the alkyl side-chain length exhibits a similar effect on the potential protein targets of polyphenols from the α-pyrone cluster as the number of hydroxyl groups in the case of polyphenols from the flavonol and flavanone clusters.

Polyphenolic compounds within the same class exhibit highly similar inverse molecular docking fingerprints due to only slight variations in their chemical structures, while between different classes, distinct protein target-specific binding patterns can be observed. Our findings demonstrate that subtle structural differences between polyphenolic compounds from flavonol, flavanone, and α-pyrone classes result in significant differences in their affinity to protein targets, suggesting that in-depth studies of the mechanisms of action of individual polyphenols from different classes are required. In this study, an inverse molecular docking fingerprint clustering approach was applied to all human protein targets; however, it can also be applied to a smaller protein set or to a specific set of protein targets from a different organism [41]. The inverse molecular docking fingerprint methodology based on hierarchical clustering, therefore, represents an innovative approach to identify compounds with similar binding patterns to various protein targets. Moreover, the method can be extended to hypothesis generation by studying the compounds and examining their potential protein targets with the best docking scores.

### 2.2. Pharmacological Relevance of Protein Targets Obtained with Inverse Molecular Docking Fingerprints

**Figure 3:**
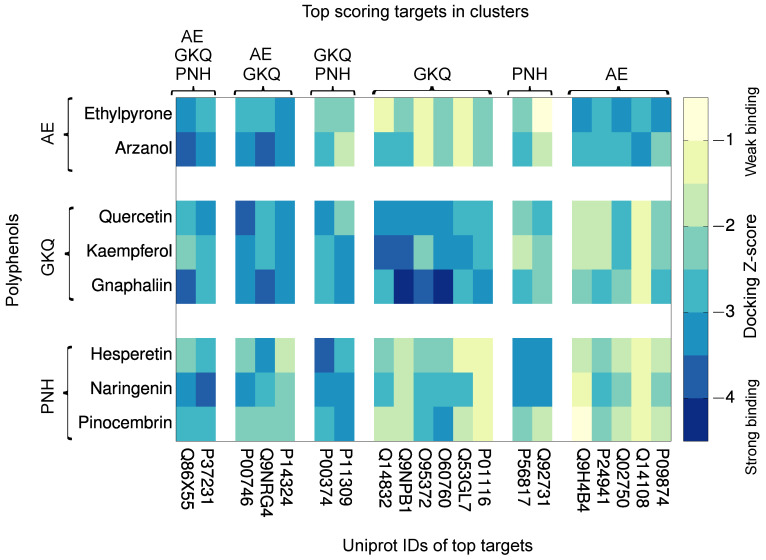
Inverse molecular docking fingerprint heatmap of investigated polyphenolic compounds. Presented protein targets exhibit an average molecular docking Z-score across all polyphenols in a fingerprint cluster (AE, GKQ, PNH, see Figure 2) below the cutoff (<−2.807, 99.75th percentile) with at least one of the clusters. For readability, the protein targets are separated by which polyphenol cluster(s) exhibit molecular docking Z-scores below the cutoff. Dark blue regions correspond to favorable docking Z-scores, indicating stronger binding, while light yellow regions correspond to higher Z-scores, indicating weaker binding. Abbreviations: AE: arzanol and ethylpyrone, GKQ: gnaphaliin, kaempferol, and quercetin, PNH: pinocembrin, naringenin, and hesperetin.

To compare the human protein targets of the eight studied *Helichrysum italicum* polyphenols, the results of the hierarchical clustering of inverse molecular docking fingerprints were applied. In order to improve our previous methodology [41], top-scoring protein targets were identified by the average docking Z-score across a cluster of polyphenols, to study specific as well as potential synergistic biological effects. Protein targets that feature at least one average docking Z-score below the selected cutoff (−2.807, 99.75th percentile) were considered, resulting in 20 top-scoring targets, which were further studied in detail. The docking Z-scores of the investigated polyphenols with the top-scoring protein targets are illustrated in Figure 3, visually depicting the inverse molecular docking fingerprint methodology.

From the inverse molecular docking fingerprint heatmap, it can be observed that the fingerprints are indeed unique for each investigated polyphenol, and that there is a diversity in molecular docking Z-score values to protein targets across different clusters. However, a clear similarity in the Z-score values to specific protein targets can be seen for polyphenols within the three different clusters. Moreover, common protein targets of polyphenols from different clusters can also be identified.

The biological and therapeutic relevance of binding sites of top-scoring protein targets was assessed by their corresponding scores using DoGSiteScorer [53]. All identified protein targets demonstrated high estimated drug scores of the corresponding docked binding sites (0.74–0.87), and their biological effects, drug target relevance, known inhibitors, and proposed roles in various diseases are collected in Table 1 and Table 2 and comprehensively discussed in the following subsections.

#### 2.2.1. Pharmacological Relevance of Potential Protein Targets Common to Multiple Polyphenol Classes

**Figure 4:**
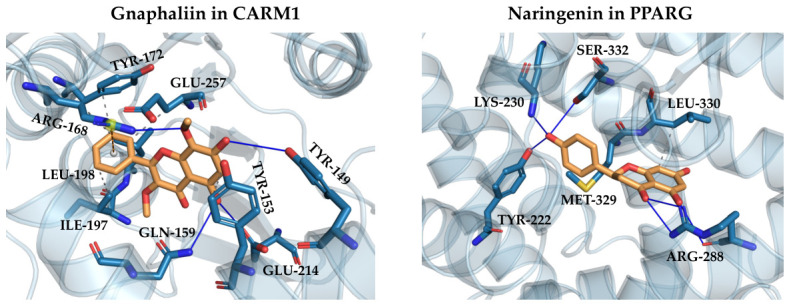
Representative docked poses of gnaphaliin in CARM1 and naringenin in PPARG. Oxygen atoms are presented in red, carbon atoms of polyphenols are orange, and carbon atoms of amino acids are depicted in cyan. Dashed grey lines denote hydrophobic interactions, blue lines represent hydrogen bonds, and orange lines indicate π–cation interaction, as determined by PLIP [54].

As can be observed from Figure 3 and Table 1, two proteins, namely, peroxisome proliferator-activated receptor gamma (PPARG) and histone-arginine methyltransferase (CARM1), exhibited very favorable docking Z-scores with polyphenols from all three clusters as well as high druggability scores (0.82 and 0.81, respectively). This may indicate promising synergistic anticarcinogenic, antiinflammatory, and antidiabetic effects among the eight investigated polyphenolic compounds from *Helichrysum italicum*. Figure 4 illustrates the intermolecular interactions (docking poses) corresponding to the most favorable docking scores of gnaphaliin in CARM1 and naringenin in PPARG. Additional information on the binding interactions of all polyphenols with CARM1 and PPARG is included in the Supplementary Materials (Section S3).

**Table 1:** Pharmacological relevance of identified top-scoring protein targets of *Helichrysum italicum* polyphenols common to multiple polyphenol classes.

Uniprot ID	Protein Name	Polyphenol Classes ^1^	Drug Score ^2^	Correlation with Disease	Known Drugs/ Polyphenolic Inhibitors
Q86X55	Histone-arginine methyltransferase (CARM1)	GKQ, PNH, AE	0.81	Breast, prostate, and colorectal cancer, as well as multiple myeloma [55]	GSK3368715 (EPZ015938) for cancer [56] (Phase I clinical trial) and EZM2302 (GSK3359088) for multiple myeloma [55] (preclinical trials)
P37231	Peroxisome proliferator-activated receptor gamma (PPARG)	GKQ, PNH, AE	0.82	Type 2 diabetes, atherosclerosis, nonalcoholic steatohepatitis (NASH) fatty liver disease, and cancer [57]	Pioglitazone for diabetes type II [58]; kaempferol, quercetin [59] and naringenin [60]
P00746	Complement factor D (CFD)	GKQ, AE	0.86	Paroxysmal nocturnal haemoglobinuria, atypical haemolytic uraemic syndrome, and macular degeneration [61]	Danicopan for paroxysmal nocturnal haemoglobinuria (Phase III trials) [62]
Q9NRG4	N-lysine methyltransferase (SMYD2)	GKQ, AE	0.81	Oesophageal, liver, and breast cancer [63], cardiovascular diseases [64]	AZ505, LLY-507, BAY-598 for cancer at the preclinical stage [63]
P14324	Farnesyl pyrophosphate synthase (FPPS)	GKQ, AE	0.81	Bone diseases (osteoporosis, Paget’s disease, and bone metastases) [65]	Bisphosphonates alendronate, risedronate, ibandronate [66] and zoledronic acid [67] for osteoporosis
P00374	Dihydrofolate reductase (DHFR)	GKQ, PNH	0.81	Leukaemia, lymphomas, cancer, rheumatoid arthritis, psoriasis (methotrexate) [68]	Methotrexate for acute leukaemias, osteosarcoma, and rheumatoid arthritis [69] (approved drug); glycoside naringin and quercetin glucuronides [70]
P11309	Serine/threonine-protein kinase 1 (PIM-1)	GKQ, PNH	0.82	Acute myeloid leukaemia, diffuse large B-cell lymphoma, and prostate cancer [71]	PIM447 (LGH447) for multiple myeloma [72] (Phase I/II trials); quercetin and naringenin [73]

^1^ Polyphenol classes (see Figure 2) for which the protein target exhibits a favorable average docking Z-score; ^2^ Drug score calculated for the identified protein binding site using the DoGSiteScorer [53] web server.

CARM1 represents a protein arginine methyltransferase type 1, which methylates histone H3 (Arg17 and Arg26) as well as various non-histone proteins involved in transcription, RNA splicing, and DNA repair [74]. CARM1 is often overexpressed in breast, prostate, and colorectal cancers, as well as in multiple myeloma, functioning as a co-activator of oncogenic transcriptional pathways [55]. CARM1 represents an oncogenic target, as its overexpression results in tumor progression. In multiple myeloma, CARM1 activates malignant plasma cell growth. Therefore, genetic or pharmacological inhibition of CARM1 leads to a reduced tumor cell proliferation. Consequently, CARM1 represents an important therapeutic target in epigenetically driven cancers. There are no approved CARM1-selective drugs yet; however, CARM1 inhibitors GSK3368715 (EPZ015938) were tested in Phase I clinical trial for cancer [56] and EZM2302 (GSK3359088) in preclinical trials for multiple myeloma [55]. To the best of our knowledge, this is the first time that natural polyphenolic compounds have been shown to potentially modulate CARM1.

PPARG represents a nuclear hormone receptor that acts as a key regulator of adipogenesis, lipid metabolism, and insulin sensitivity. It forms a validated therapeutic target in metabolic diseases, such as type 2 diabetes, as activation of PPARG improves insulin sensitivity and glucose uptake. PPARG also modulates inflammation and differentiation in macrophages, making it an important target in conditions such as atherosclerosis, nonalcoholic steatohepatitis (NASH) fatty liver disease, and cancer. In adipose tissue, PPARG promotes fat cell differentiation and lipid storage, while in immune cells, it can exert antiinflammatory effects. Consequently, PPARG represents an important therapeutic target in diabetes, metabolic syndrome, and potentially in inflammation-related diseases, such as cancer [57]. PPARG agonist pioglitazone is an approved drug for type 2 diabetes [58], which improves glycaemic control by increasing insulin sensitivity. On the other hand, the use of rosiglitazone declined after concerns regarding cardiovascular risks. Beyond diabetes, PPARG agonists have been investigated in NASH, polycystic ovary syndrome, and cancer; however, no PPARG agonist has been approved due to adverse side effects such as fluid retention, weight gain, and bone loss. Several natural products were also reported to exhibit PPARG activation. For example, flavonols kaempferol and quercetin, as well as diterpenes carnosic acid and carnosol from *Rosmarinus officinalis*, were reported as PPARG activators [75]. Additionally, flavanone naringenin was also demonstrated as a PPARG agonist [60], which is in agreement with the obtained results in our computational study.

High-scoring proteins, namely, dihydrofolate reductase (DHFR) and serine/threonine-protein kinase 1 (PIM-1), which are also involved in cancer cell proliferation, represent potential targets of both flavonol-class as well as flavanone-class polyphenols.

PIM-1 is a proto-oncogene serine/threonine kinase that activates cell survival and proliferation. It is often overexpressed in haematological cancers such as acute myeloid leukaemia and diffuse large B-cell lymphoma, as well as in solid tumors, such as prostate cancer [71]. PIM-1 enhances tumor cell survival by phosphorylating proteins involved in apoptosis (e.g., BCL2-associated agonist of cell death (BAD) protein) and the cell cycle (e.g., cyclin-dependent kinase inhibitor 1), as well as contributes to drug resistance. As PIM-1 is not essential in healthy cells due to functional redundancy with PIM-2 and PIM-3, it is an attractive anti-cancer target, particularly in tumors with high PIM expression or JAK/STAT activation. Inhibiting PIM-1 induces apoptosis and cell cycle arrest in cancer cells, supporting its validity as an important target in oncology. No PIM kinase inhibitor has received full approval yet; however, some candidates have entered clinical trials. PIM447 (LGH447) represents the most clinically advanced PIM inhibitor that has been tested in Phase I/II trials for multiple myeloma, demonstrating good tolerability [72]. SGI-1776 reached Phase I trials in lymphoma; however, it was terminated due to QT prolongation [76]. Moreover, flavonol quercetin and flavanone naringenin have been reported to exhibit PIM1 inhibition in silico and in vitro (IC_50_ values 34.9 μM and 28.6 μM, respectively), demonstrating their potential in prostate cancer treatment [73].

DHFR is a key enzyme in folate metabolism, which is required for regenerating tetrahydrofolate in DNA synthesis. It represents an important therapeutic target in rapidly proliferating cancer cells. DHFR is targeted to inhibit thymidine production and DNA replication, which is crucial for cancer chemotherapy in leukaemia and lymphomas. Human DHFR is also targeted in autoimmune diseases via immunosuppressive antifolates. Therefore, DHFR represents an important protein target for cancer treatment, rheumatoid arthritis, psoriasis (methotrexate), as well as for bacterial or protozoal infections [68]. DHFR is one of the most clinically exploited targets, with several approved inhibitors. Its inhibitors are widely used in oncology and anti-infective therapy. For example, methotrexate represents an anchor drug in chemotherapy for acute leukaemias and osteosarcoma as well as in immunosuppressive therapy for the treatment of rheumatoid arthritis [69]. Moreover, an antibiotic, trimethoprim (with sulfamethoxazole) targets bacterial DHFR for the treatment of urinary and respiratory infections. Notably, leucovorin (folinic acid) is co-administered to mitigate the toxic effects of DHFR inhibition in normal cells [77]. Glycoside naringin as well as quercetin glucuronides were reported to inhibit DHFR, while their binding affinity was highly dependent on the sugar residue [70]. Our computational results also elucidated that flavanones and flavonols from *Helichrysum italicum* present potential DHFR modulators; however, further experimental studies are needed to confirm their binding affinity.

Favorable docking Z-score values to three protein targets, namely, N-lysine methyltransferase (SMYD2), farnesyl pyrophosphate synthase (FPPS), and complement factor D (CFD), were also observed in both flavanone and α-pyrone clusters, indicating possible synergistic effects between gnaphaliin, quercetin, kaempferol, arzanol, and ethylpyrone. The inhibition of these proteins leads to reduced cancer cell proliferation and survival.

SMYD2 is a histone lysine methyltransferase that methylates both histone, such as H3K36, and non-histone substrates, including p53, retinoblastoma protein (Rb), estrogen receptor alpha (ERα), and poly [ADP-ribose] polymerase 1 (PARP1). It is overexpressed in many cancer types and can methylate tumor suppressors, such as p53, to reduce their activity. SMYD2 represents a promising oncogenic target in cancers, including oesophageal, liver, and breast cancer, as its inhibition suppresses tumor cell growth and proliferation [63]. SMYD2 also plays a role in cardiovascular diseases and inflammation [64]. Currently, there are no approved drugs targeting SMYD2, although known inhibitors, namely, AZ505, LLY-507, and BAY-598, with promising results at the preclinical stage, have been reported [63]. SMYD2 is being investigated for cancer therapy to reactivate p53 or block pro-cancer gene expression; however, specificity and off-target effects continue to raise concerns. In summary, SMYD2 represents a promising but still experimental oncology target, with ongoing research focused on developing more potent and safer inhibitors. To date, polyphenolic compounds have not been reported as potent SMYD2 inhibitors in the scientific literature, and the data on SMYD2 binding are still lacking. Our results elucidated, for the first time, the potential polyphenolic modulators of SMYD2, which should be further explored in experimental studies.

FPPS represents a key enzyme in the mevalonate pathway, catalyzing the synthesis of farnesyl pyrophosphate, a precursor of cholesterol, steroid hormones, and isoprenoids. Importantly, FPPS is also required for protein farnesylation or geranylgeranylation of small GTPases, such as Ras and Rho [65]. Inhibition of FPPS leads to downstream effects on bone metabolism and tumor cell survival. The main therapeutic relevance of FPPS lies in bone diseases, as inhibiting FPPS in osteoclasts induces apoptosis and retains bone resorption. Therefore, FPPS represents the target of bisphosphonate drugs used in osteoporosis, Paget’s disease, and bone metastases. indirectly, it is also considered in cancer therapy, as blocking prenylation can impair oncogenic Ras function. Overall, FPPS is targeted for bone density preservation and anti-tumor effects in bone. FPPS inhibitors are well-established and widely approved drugs for osteoporosis with a strong safety and efficacy profile for bone protection. For example, bisphosphonates alendronate, Risedronate, and Ibandronate are oral agents for osteoporosis that reduce fracture risk by inhibiting osteoclast FPPS and inducing osteoclast apoptosis [66]. Zoledronic acid is an intravenous bisphosphonate used for osteoporosis (administered yearly) and for preventing breast or prostate cancer bone metastasis and multiple myeloma [67]. In summary, FPPS is a validated clinical target, and bisphosphonates are the prototypical FPPS-targeted drugs, essential in both osteoporosis management and anti-cancer therapies. The natural compounds taxodione (a diterpene quinone from yew) and arenarone (a marine sesquiterpene) have been reported to inhibit mammalian FPPS by interacting with the same substrate binding site as bisphosphonates [78]. However, no flavonoids or α-pyrones with potent FPPS activity are reported in the scientific literature. Therefore, potential polyphenolic FPPS modulators were reported for the first time in this computational study and should be further explored.

CFD, another high-scoring protein target, is a serine protease essential for the alternative pathway of the complement system. Overactivation of this pathway is related to diseases such as paroxysmal nocturnal haemoglobinuria, atypical haemolytic uraemic syndrome, and age-related macular degeneration. CFD is the rate-limiting enzyme in the alternative complement cascade, representing an attractive therapeutic target to reduce complement-mediated inflammation. Inhibiting factor D is proposed to prevent harmful complement amplification in autoimmune, inflammatory, and age-related diseases [61]. There are no approved CFD inhibitors yet; however, danicopan is in Phase III trials as an add-on therapy in paroxysmal nocturnal haemoglobinuria to reduce extravascular haemolysis [62]. There are also some related safety concerns, as chronic complement suppression can increase infection risks [61]. Natural polyphenols have not yet been reported to inhibit CFD in the scientific literature. Our results indicate that CFD is a potential target of flavonol and α-pyrone class polyphenols.

#### 2.2.2. Pharmacological Relevance of Potential Protein Targets Unique to a Single Polyphenol Class

Moreover, unique protein targets for each polyphenol class were also identified (Figure 3 and Table 2). Flavanone class polyphenols naringenin, pinocembrin, and hesperetin demonstrated high affinity to two protein targets, namely, beta-secretase 1 (BACE1) and estrogen receptor beta (ERβ).

**Table 2:** Pharmacological relevance of identified top-scoring protein targets of *Helichrysum italicum* polyphenols unique to a single polyphenol class.

Uniprot ID	Protein Name	Polyphenol Classes ^1^	Drug Score ^2^	Correlation with Disease	Known Drugs/ Polyphenolic Inhibitors
Q14832	Metabotropic glutamate receptor 3 (GRM3)	GKQ	0.79	Schizophrenia, anxiety and bipolar disorder [79]	Pomaglumetad methionil for schizophrenia and anxiety [80] (Phase II/III trials)
Q9NPB1	5′(3′)-Deoxyribonucleo-tidase	GKQ	0.80	Cancer, acute lymphoblastic leukaemia [81]	CRCD2 for acute lymphoblastic leukaemia [82]
O95372	Acyl-protein thioesterase 2 (APT2)	GKQ	0.81	Pancreatic cancer, melanomas and neurological disorders [83]	ML349 for cancer (preclinical trials) [83]
O60760	Hematopoietic prostaglandin D synthase (HPGDS)	GKQ	0.84	Asthma, allergic rhinitis, atopic dermatitis, atherosclerosis and CNS inflammation [84]	HQL-79 for allergic and inflammatory conditions [85] (preclinical stage); quercetin [86] (in silico)
Q53GL7	Poly [ADP-ribose] polymerase 10 (PARP10)	GKQ	0.74	Cancer and immune response [87]	OUL35 for cancer [88] (preclinical studies)
P01116	GTPase Kras (KRas)	GKQ	0.82	Non-small cell lung and colon cancer [89]	Sotorasib [90] and adagrasib [91] for non-small cell lung cancer (NSCLC); quercetin [92] (indirect indication)
P56817	Beta-secretase 1 (BACE1)	PNH	0.81	Alzheimer’s disease [93]	Verubecestat [94] and atabecestat [95] for Alzheimer’s disease (terminated in Phase III trials); hesperetin and naringenin [96]
Q92731	Estrogen receptor beta (ERβ)	PNH	0.84	Breast and prostate cancer, menopausal symtomps [97]	Diarylpropionitrile for anxiolytic and antidepressant effects [98] (preclinical studies); naringenin [99]
Q9H4B4	Serine/threonine-protein kinase (PLK3)	AE	0.82	Cancer, heart attack and stroke [100]	No
P24941	Cell division protein kinase 2 (CDK2)	AE	0.81	Breast and ovarian cancers [101]	Roscovitine (seliciclib) for lung cancer (Phase II clinical trials) [102]
Q02750	Dual specificity mitogen-activated protein kinase 1 (MEK1)	AE	0.81	Melanoma and lung cancer [103]	Trametinib for the treatment of BRAF V600-mutant melanoma [104] and for metastatic BRAF-mutant thyroid cancers [105], cobimetinib and binimetinib for BRAF-mutant melanoma [106,107]
Q14108	Lysosome-associated membrane protein 2 (LAMP2)	AE	0.81	Danon disease [108]	Arzanol (indirect indication) [109]
P09874	Poly [ADP-ribose] polymerase 1 (PARP1)	AE	0.81	Melanomas, breast, ovarian, prostate, and lung cancers [110], Alzheimer’s, Parkinson’s, and Huntington’s disease [111]	Olaparib, rucaparib, niraparib, and talazoparib for ovarian, breast, prostate, and pancreatic cancers [112]; resveratrol [113] and curcumin [114]

^1^ Polyphenol classes (see Figure 2) for which the protein target exhibits a favorable average docking Z-score; ^2^ Drug score calculated for the identified protein binding site using the DoGSiteScorer [53] web server.

BACE1 is an aspartic protease responsible for the cleavage of amyloid precursor protein (APP), leading to the production of amyloid-β peptide, a key building block in the pathogenesis of Alzheimer’s disease (AD). BACE1 activation results in the amyloid-β accumulation, which is a hallmark of AD. Therefore, BACE1 has been a primary target in AD therapy, as BACE1 inhibition reduces Aβ production, potentially slowing or preventing the progression of Alzheimer’s disease [93]. BACE1 is also involved in myelination and may play roles in diabetic neuropathy and retinal degeneration due to its cleavage of neuregulin. However, the therapeutic focus has been on AD to combat neurodegeneration by reducing the amyloid levels. Despite the invested effort, no BACE1 inhibitor has yet been approved. Some inhibitors that advanced to Phase III trials in Alzheimer’s disease, namely, verubecestat (Merck) [94] and atabecestat [95], but were terminated due to the worsening of cognitive tests and their adverse side effects, such as liver toxicity. The research should, therefore, focus on testing BACE inhibitors in earlier (pre-symptomatic) stages of Alzheimer’s disease and adjusting the dose to avoid these side effects. Several natural compounds have been reported to inhibit BACE1 in vitro, although with much lower potency than synthetic drugs. Polyphenols were shown to weakly inhibit BACE1 and to provide structural insights for drug design. For example, hesperetin (${\mathrm{IC}}_{50}=22.13\pm 1.81$ μM) and naringenin (30.31±2.06 μM) demonstrated promising inhibitory properties toward BACE1 in the micromolar range [96]. The results of this study also demonstrated that polyphenols with a flavanone core represent potential BACE1 modulators that could be further modified to yield more effective BACE1 inhibitors with fewer side effects.

ERβ is one of the two main estrogen receptor subtypes (ERα and ERβ) that represent ligand-activated transcription factors. ERβ is expressed in many tissues, including the ovary, prostate, central nervous and cardiovascular systems. ERβ controls anti-proliferative and pro-differentiation effects in cells. In breast cancer, ERβ activation may inhibit tumor growth as it is considered a tumor suppressor [115]. In prostate cancer, ERβ agonists might suppress proliferation and induce apoptosis, as ERβ is the predominant ER in healthy prostate cells. Selective ERβ agonists are being investigated for relief of menopausal symptoms, such as hot flushes, without stimulating breast or uterine tissues. Therefore, ERβ-selective agonists represent potential candidates to treat cognitive, mood, or vasomotor symptoms in menopause, osteoporosis through beneficial effects on bones, and colorectal cancer or triple-negative breast cancer [97]. The goal is to maintain the beneficial effects of estrogen via ERβ while avoiding ERα-driven side effects, such as breast or uterine proliferation. Currently, there are no approved ERβ-specific modulators on the market. However, some ERβ agonists have entered clinical trials. For example, diarylpropionitrile (DPN) demonstrated ≈70-fold selectivity for ERβ over Erα. Preclinical studies with diarylpropionitrile have shown anxiolytic and antidepressant effects in vivo [98]. One challenge is achieving complete selectivity, and the long-term effects of systemic ERβ agonism remain uncertain. The field continues to actively search for new compounds with improved clinical effects. Polyphenolic compounds have also demonstrated relative selectivity for ERβ. For example, genistein from soya beans binds to ERβ approximately 7–30 times more strongly than it does to ERα [97]. Moreover, naringenin was reported to specifically activate ERβ, which improved insulin secretion in the primary rat islets [99]. The results of our computational study also demonstrated that flavanones represent potential modulators of ERβ. Therefore, flavanone-class polyphenols should be explored in future experimental studies and could be further modified for pharmaceutical development.

Furthermore, flavanones gnaphaliin, kaempferol, and quercetin demonstrated high affinity to specific protein targets, namely, poly [ADP-ribose] polymerase 10 (PARP10), metabotropic glutamate receptor 3 (GRM3), 5′(3′)-deoxyribonucleotidase, acyl-protein thioesterase 2 (APT2), GTPase KRas (KRas), and hematopoietic prostaglandin D synthase (HPGDS).

PARP10 is a mono-ADP-ribosyltransferase that adds single ADP-ribose units to target proteins (MARylation). It is involved in various cellular processes, including DNA damage tolerance, replication stress response, and cell proliferation [87]. PARP10 has been reported to promote the restart of stalled replication forks in the nucleus and to reduce replication stress in cells. It also negatively regulates oncogenic signalling pathways by MARylation. Overexpression of PARP10 can facilitate tumor growth and chemotherapy resistance by promoting DNA repair and replication [87]. PARP10 overexpression can, on the other hand, cause cell death through its catalytic activity [88]. In cancer, inhibiting PARP10 might reduce resistance of tumor cells to DNA-damaging therapies and prevent recovery from replication stress [116]. PARP10 also plays a role in immunity, as inhibiting PARP10 in tumor-associated macrophages can trigger inflammatory responses [117]. Therefore, PARP10 represents an important protein target in oncology, which regulates genotoxic stress and modulates immune cancer cells. To the best of our knowledge, there are currently no PARP10-specific inhibitors in clinical trials or in clinical use. For now, PARP10 inhibitors, such as OUL35, are applied solely in preclinical studies to validate PARP10 as a viable target in certain tumor models [88]. Therefore, our computational study for the first time elucidated that polyphenols from the flavanone class represent potential PARP10 modulators, as no polyphenol or natural products have yet been reported to inhibit PARP10.

GRM3 is a group II metabotropic glutamate receptor that modulates glutamate neurotransmission in neurons and has been connected with psychiatric and neurological disorders. Genetic studies reported GRM3’s involvement with schizophrenia and bipolar disorder. Its dysfunction is implicated in cognitive impairment, as it produces antipsychotic effects by reducing excessive glutamate release [79]. Consequently, GRM3 is considered a therapeutic target for schizophrenia, anxiety, and neurodegenerative diseases involving glutamate dysregulation. To date, no GRM3-specific drug has been approved. However, group II metabotropic glutamate receptor agonists, such as pomaglumetad methionil, are being investigated in Phase II/III clinical trials [80]. No polyphenolic compounds have yet been reported to selectively bind to GRM3. The results of our computational study demonstrated that flavanone-class polyphenols potentially bind to GRM3 with high affinity, which should be explored in future experimental studies. Overall, GRM3 represents a validated but challenging central nervous system protein target. Further research is needed to develop safer, more effective GRM3-targeted therapies for cognitive enhancement in schizophrenia.

The enzyme 5′(3′)-Deoxyribonucleotidase dephosphorylates deoxyribonucleoside monophosphates to nucleosides. It controls intracellular dNTP pools and the metabolism of nucleoside analogue drugs by removing the phosphate group, which limits the drug’s efficacy. Therefore, 5′(3′)-deoxynucleotidase is considered a protein target to overcome drug resistance in cancer and to modulate dNTP balance in metabolic disorders [81]. It is also involved in nucleotide homeostasis by preventing an imbalance that can cause DNA instability. There are currently no approved drugs for targeting 5′(3′)-deoxyribonucleotidase; however, their potential lies in chemotherapy against acute lymphoblastic leukaemia (ALL). Inhibitors, such as CRCD2, have shown promising results but did not enter clinical trials for ALL with cytosolic 5′-nucleotidase II (NT5C2) mutations [82]. No specific polyphenolic inhibitors of 5′(3′)-deoxyribonucleotidase have been reported yet. According to our results, flavanone-class polyphenols could directly modulate 5′(3′)-deoxyribonucleotidase pathway, which opens new research opportunities.

APT2 is an enzyme that cleaves thioester-linked fatty acyl chains (palmitate) from cysteine protein residues. APT1 and APT2 modulate the localisation and function of oncogenic proteins HRAS and NRAS, which require cycles of palmitoylation for their proper function. By removing palmitate, APT2 can induce another round of palmitoylation of RAS. In Ras-driven cancers, such as pancreatic cancer and melanomas, inhibiting APT1/2 leads to mislocalisation of oncogenic Ras and the suppression of tumor growth [83]. APT2 has also been implicated in neuronal function by depalmitoylating synaptic proteins such as GAP-43 and impacting axonal growth, which could affect synaptic plasticity [118]. Therefore, APT2 is being investigated as a target in both Ras-driven cancers and neurological disorders. For example, in NRAS-mutant melanoma, inhibiting APT1 and APT2 was found to mislocalise NRAS and impair signaling [119]. No APT2-specific inhibitors were tested in clinical trials. However, APT2 inhibitors, such as ML349, are being investigated in preclinical cancer models [83]. Polyphenols have not been reported to specifically inhibit APT2 yet. Sulforaphane is the only example of a natural APT2 inhibitor [120]. Therefore, the results of our computational study for the first time demonstrated that flavonol-class polyphenols could directly inhibit APT2. Currently, APT2 remains a research-stage target, with potential therapeutic applications in oncology and neurological disorders.

KRas is a small GTPase that represents a molecular switch in MAPK and PI3K signalling pathways. KRAS mutations occur in approximately 25% of cancers, including pancreatic adenocarcinomas, colorectal cancers, and lung adenocarcinomas [89]. KRas was considered as “undruggable” target due to its picomolar affinity for Guanosine triphosphate (GTP) and Guanosine diphosphate (GDP). However, a specific mutant KRAS G12C was recently targeted with covalent inhibitors. In oncology, KRas is considered an important protein target, specifically for tumors with mutant KRAS. Inhibiting KRas blocks downstream RAF/MEK/ERK, AKT/mTOR, and RAL-GDS signalling pathways, leading to tumor regression [89]. There are two approved KRAS G12C inhibitors, which represent the first direct Ras inhibitors after nearly 40 years of research. Sotorasib (2021) is approved for non-small cell lung cancer (NSCLC) with an acceptable safety profile with mild gastrointestinal symptoms and liver enzyme elevations [90]. Adagrasib (2022) was also approved for KRAS G12C-mutant NSCLC treatment after chemotherapy or immunotherapy [91]. Flavanone quercetin has been reported to reduce KRas activity in colon cancer cells [92]; however, it was not reported to directly bind to KRas. Results of our study demonstrated that flavanone-class polyphenols quercetin, gnaphaliin, and kaempferol could potentially directly bind to KRas with high affinity.

HPGDS is an enzyme which catalyzes the conversion of PGH_2_ to prostaglandin D_2_ (PGD_2_) and is expressed in mast cells, basophils, and Th2 lymphocytes. PGD_2_ represents a key mediator in allergic and inflammatory responses and plays an important role in asthma, allergic rhinitis, and atopic dermatitis [84]. Inhibiting HPGDS reduces the production of PGD_2_, which can reduce allergic inflammation, including bronchoconstriction and inflammatory response in asthma. HPGDS has also been implicated in atherosclerosis and CNS inflammation. HPGDS is, therefore, considered a therapeutic target for inflammatory diseases, such as allergic asthma and nasal allergies. Currently, there are no HPGDS inhibitors available on the market. HPGDS inhibitors, such as HQL-79, remain at the preclinical stage [85]. However, HPGDS represents a promising protein target for allergic and inflammatory conditions. In experimental studies, dietary polyphenols, such as resveratrol and quercetin, have demonstrated antiinflammatory effects that could indirectly reduce PGD_2_ levels [121]. Quercetin was indeed proposed to exert potential antiallergic and antiinflammatory activity by direct inhibition of HPGDS in in silico study [86]. This is in good agreement with our results, which demonstrated that flavanone-class polyphenols represent potential HPGDS inhibitors.

Furthermore, in this study the potential protein targets of α-pyrone-class compounds arzanol and ethylpyrone were identified, namely, poly [ADP-ribose] polymerase 1 (PARP1), dual specificity mitogen-activated protein kinase 1 (MEK1), lysosome-associated membrane protein 2 (LAMP2), cell division protein kinase 2 (CDK2), and serine/threonine-protein polo-like kinase 3 (PLK3).

PARP1 is a nuclear enzyme that plays an important role in maintaining genomic integrity by repairing single-strand and double-strand DNA breaks. It catalyzes poly (ADP-ribosyl)ation using NAD^+^, modifies chromatin structure, and recruits DNA repair proteins, which is essential for cell survival under genotoxic stress. PARP1 is often overexpressed in melanomas, breast, ovarian, prostate, and lung cancers. In tumors with BRCA1/2 mutations or homologous recombination deficiency, inhibition of PARP1 leads to a selective cancer cell death while normal cells remain undamaged [110]. PARP1 overactivation has also been implicated in neurodegenerative disorders such as Alzheimer’s, Parkinson’s, and Huntington’s diseases [111]. It also plays roles in cardiac injury and inflammatory conditions due to its role in chromatin remodeling and transcriptional regulation [122]. PARP1 is considered an attractive drug target, and several drugs targeting PARP1 have been developed, namely, olaparib, rucaparib, niraparib, and talazoparib [112]. All drugs were approved for BRCA-mutated ovarian, breast, prostate, and pancreatic cancers. Dietary polyphenols, such as resveratrol [113] and curcumin [114], have been reported to complement pharmacological PARP1 inhibition strategies. In addition, our results identified α-pyrone-class compounds arzanol and ethylpyrone as potential PARP1 inhibitors.

MEK1 is a dual-specificity kinase, which phosphorylates the extracellular signal-regulated kinase 1/2 (ERK1/2) in RAS/RAF/MEK/ERK signaling cascade. It plays a crucial role in cell proliferation and survival. Activation of MEK1/2 is related to the oncogenic RAS or BRAF mutations. MEK1 represents an important target in cancers, such as BRAF-mutant melanoma and KRAS-mutant lung cancer [103]. Inhibition of MEK1 prevents cell cycle progression and induction of apoptosis in tumor cells [123]. There are some approved MEK1/2 inhibitors in clinical use, which are primarily applied in combination with other drugs. Trametinib is approved for the treatment of BRAF V600-mutant melanoma in combination with dabrafenib [104], and for metastatic BRAF-mutant thyroid cancers [105], while cobimetinib and binimetinib are approved for BRAF-mutant melanoma in combination with vemurafenib [106] and encorafenib [107]. However, these inhibitors exhibit adverse effects such as rash, diarrhoea, retinal vein occlusion, and cardiomyopathy. In the scientific literature, natural compounds have not yet been reported to directly inhibit MEK1/2. The results of our computational study for the first time demonstrated that α-pyrone-class natural compounds arzanol and ethylpyrone could potentially inhibit MEK1 activity.

LAMP2 is a glycoprotein located on lysosomal membranes that plays a role in chaperone-mediated autophagy (CMA) and lysosomal function. LAMP2 mutations cause Danon disease, a rare X-linked disorder, characterised by cardiomyopathy, myopathy, and intellectual disability due to impaired lysosomal degradation [108]. Due to LAMP2 deficiency in Danon disease, the upregulation of LAMP2 is desired, as it leads to the accumulation of autophagic vacuoles in heart and muscle tissues. LAMP2 modulation may be relevant in neurodegenerative diseases, as enhancing CMA leads to clearance of alpha-synuclein or tau proteins. Therefore, LAMP2 could be targeted as a regulator of autophagy in chronic diseases. No synthetic or natural compound is reported to directly inhibit LAMP2. Arzanol was indeed reported to induce autophagy [109], which could partially compensate for mild LAMP2 insufficiency. According to our results, the molecular mechanism of the observed effects could lie in arzanol’s inhibition of LAMP2.

CDK2 is a cyclin-dependent kinase that is involved in the G1-S phase of the cell cycle and is essential for the initiation of DNA replication. CDK2 is overexpressed in many cancers, including aggressive breast and ovarian cancers, which often exhibit overexpression of cyclin E, leading to CDK2 hyperactivation. CDK2 represents an important drug target that has been investigated in cancer therapy to modulate uncontrolled proliferation in tumors with an intact Rb pathway [101]. In recent years, CDK2 has represented an important target in cyclin E-overexpressing breast cancers to induce cell cycle arrest or to overcome their resistance to CDK4/6 inhibitors. No CDK2-specific inhibitor has been approved yet; however, some CDK2 inhibitors have entered clinical trials. For example, roscovitine (seliciclib) reached Phase II clinical trials in lung cancer but did not progress further, due to limited potency and unspecific CDK inhibition [102]. However, CDK2 inhibitors are faced with toxicity challenges due to the inhibition of various CDKs in normal tissues. In addition, our results for the first time demonstrated that α-pyrone-class compounds arzanol and ethylpyrone could potentially modulate CKD2, which should be further investigated in experimental assays.

PLK3 is involved in cell cycle regulation and stress response. PLK3 is considered a tumor suppressor, which is activated by DNA damage and hypoxia, mediating cell cycle arrest or apoptosis in response to genotoxic stress [124]. PLK3 participates in the DNA damage checkpoint and can promote phosphorylation of p53 and cell division cycle 25C (CDC25C), which inhibits cell cycle progression. PLK3 also negatively regulates angiogenesis by destabilizing the hypoxia inducible factor-1 (HIF-1α) through phosphorylation of siah E3 ubiquitin protein ligase 2 (SIAH2) and stabilization of the phosphatase and tensin homolog (PTEN) [125]. The downregulation of PLK3 activity has been associated with increased tumor angiogenesis and growth [125]. PLK3 supports normal stress responses and prevents uncontrolled cell proliferation, therefore, PLK3 inhibition may promote the survival of cancer cells. In oncology, the aim is to activate PLK3, as it is considered a tumor suppressor [126]. PLK3 inhibition could be beneficial in organ transplantation, heart attack or stroke, where preventing PLK3-induced apoptosis is beneficial [100]. Currently, there are no reported drugs or natural compounds that specifically target PLK3. Our inverse molecular docking results indicate that α-pyrone-class compounds arzanol and ethylpyrone represent potential PLK3 modulators.

### 2.3. Discussion

In the scientific literature, there is limited evidence of interactions between the identified high scoring protein targets and investigated polyphenolic compounds from flavonol, flavanone, and α-pyrone classes. In this study, the inverse molecular docking fingerprint approach based on hierarchical clustering was introduced to analyze the binding patterns of flavonol, flavanone, and α-pyrone-class polyphenols from *Helichrysum italicum*, which resulted in three clusters, indicating similarities and differences in target protein binding patterns. Our results, indeed, represent an important contribution to improving the mechanistic understanding of anticarcinogenic and antiinflammatory effects of studied *Helichrysum italicum* polyphenols from structurally different classes. The majority of anticancer effects were reported for flavonol and flavanone-class polyphenols, while α-pyrone-class compounds arzanol and ethylpyrone remain unexplored [127]. Top-scoring protein targets, identified by our inverse molecular docking fingerprint approach, provide valuable insights into molecular mechanisms of flavonol, flavanone, and α-pyrone-class polyphenols, as well as form a firm foundation for future exploration in experimental assays.

From the top-scoring protein targets, specific binding patterns of polyphenolic compounds from flavonol, flavanone, and α-pyrone classes can be identified together with their potential synergistic effects. For the first time, synergistic effects were suggested through favorable docking scores of all eight investigated *Helichrysum italicum* polyphenols to two proteins, namely, CARM1 and PPARG, which represent important oncogenic and antidiabetic targets. Flavonol and α-pyrone-class polyphenols also demonstrated favorable docking Z-scores to three specific protein targets, namely, SMYD2, FPPS, and CFD, while flavonol and flavanone-class polyphenols exhibit potential synergistic effects through binding to DHFR and PIM-1. Unique top-scoring protein targets of flavonol, flavanone, and α-pyrone class polyphenols, related to cancer, neurodegeneration, and osteoporosis, were also identified.

The inverse molecular docking fingerprints represent a narrowed list of the most promising protein targets to investigate potential synergistic effects as well as to discover new modes of action (MOA) of *Helichrysum italicum* polyphenols in future in silico, in vitro, and in vivo studies. It is important to emphasize that the protein targets were identified by applying an arbitrary Z-score cutoff, and some relevant pharmacological targets may be excluded from the list. Therefore, the ranked lists of the top protein targets of eight investigated *Helichrysum italicum* polyphenolic compounds, namely, arzanol, ethylpyrone, gnaphaliin, quercetin, kaempferol, naringenin, pinocembrin, and hesperetin, are provided in the Supplementary Materials (Tables S20–S27). As polyphenols are known for their promiscuous binding to numerous protein targets, our results represent an important contribution by narrowing down their most promising protein targets, which have not yet been identified. Moreover, the obtained inverse molecular docking fingerprints can help rationalize molecular mechanisms of already observed biological effects of studied *Helichrysum italicum* polyphenols as well as predict their synergistic effects through identifying new and already confirmed protein targets.

Although inverse molecular docking fingerprints (Figure 2 and Figure 3) demonstrate general trends and predict potential binding patterns, further in silico, in vitro, and in vivo studies of individual polyphenols as well as their combinations are required to confirm their health-promoting effects. Molecular docking methods often prioritize speed over precision, leading to approximations in both the sampling of conformations and scoring, which may hinder the identification of true positive targets. The latter is especially critical, as target or ranking bias can be introduced by specific scoring functions. This work represents a conceptual and methodological evolution of inverse molecular docking fingerprint selection, building directly upon our previous clustering-based approaches while substantially improving the transparency and interpretability of the resulting protein target rankings. By formalizing the use of full fingerprint vectors, hierarchical clustering, and cluster-averaged Z-score–based target prioritization, the presented methodology reduces ad hoc target selection. We firmly believe that the obtained results provide valuable insight into the similarities and differences in binding patterns of flavonol, flavanone and α-pyrone classes of major polyphenols from *Helichrysum italicum* and successfully predict their potential protein targets.

## 3. Materials and Methods

### 3.1. Inverse Molecular Docking Protocol

Inverse molecular docking was performed with the software ProBiS-Dock [128] (http://insilab.org/probisdock/, accessed 15 May 2025), which employs a hierarchical approach to reconstruct small ligands in binding sites of target proteins by utilizing graph theory and generalized statistical scoring functions. In the first step, the algorithm deconstructs a given ligand into smaller fragments, which are subsequently docked into the binding sites of target proteins by applying knowledge-based scoring functions. A maximum clique algorithm and subsequent conformational optimization are applied to identify and assemble the optimal binding modes of ligand fragments at the binding site. ProBiS-Dock software also takes into account the solvent, metal ions, and interactions with metallic cofactors, which are not considered by traditional docking programs [128]. The obtained docking scores are expressed in arbitrary units. Applications of ProBiS-Dock algorithm for inverse molecular docking have been comprehensively validated by several retrospective metrics and redocking in our previous studies [38,39,40,41,128]. However, it should be taken into consideration that the ranking of ligand’s docking Z-scores in molecular docking as well as inverse molecular docking is subjected to significant limitations due to simplified statistical scoring functions. The obtained results of inverse molecular docking, therefore, correspond to an enriched set of ranked potential protein targets of eight studied polyphenols from *Helichrysum italicum*, which should be thoroughly examined in further computational or experimental studies in order to confirm their binding affinity to the predicted protein targets.

Our approach overcomes one of the main challenges of inverse molecular docking, as it limits the docking space to specific protein binding sites, which leads to reduced computational time and complexity. Locations of non-redundant small-molecule binding sites of human protein targets were obtained from the ProBiS-Dock database [37], which is constructed from 100% sequence identity clusters of proteins from the RCSB PDB [129]. In ProBiS-Dock database, small-molecule binding sites are determined by the ProBiS algorithm, based on binding site comparison [130] and further clustering of binding-site locations. The ProBiS-Dock database was successfully utilized in our previous inverse molecular docking studies [39,40,41], and has also inspired new tools for the identification of conserved water molecules and metal binding sites in protein structures [131]. Our study applies the human subset of the ProBiS-Dock database, which contains 43,269 protein structures and predetermined small-ligand binding sites. The protein structures from ProBiS-Dock database contain metal ions but exclude organic cofactors and water molecules. This limits the applicability of inverse molecular docking for organic cofactor-dependent enzymes.

In this study, eight major polyphenolic compounds from *Helichrysum italicum* were investigated, namely, α-pyrones arzanol and ethylpyrone, flavonols gnaphaliin, kaempferol, and quercetin, as well as flavanones naringenin, pinocembrin, and hesperetin (Figure 1). Optimized 3D conformations of input structures used for subsequent inverse molecular docking were prepared in Avogadro [132] and geometrically optimized with Density Functional Theory (DFT) functional M062X/6-311++G (d,p) using the Gaussian 16 software [133].

Inverse molecular docking results were analyzed by linking PDB IDs and chain identifiers with corresponding UniProt IDs, as a unique UniProt ID corresponds to a specific protein [134]. Following our previous study [41], in a case where multiple docked structures share the same UniProt ID, the lowest docking score value was used for each ligand. This grouping resulted in 3690 unique protein targets, with an average of 11.7 and a median of 3 PDB structures per unique protein target. This is a consequence of protein structures with multiple identical chains with only minor differences in sequence lengths or mutations, which were not included in the same cluster after the 100% sequence identity clustering in the ProBiS-Dock database.

As the ProBiS-dock database only includes structures from the PDB up to mid-2020 [37], future studies would benefit from the development of updated binding-site databases with a focus on highly non-redundant sets and de-duplication to reduce a possible bias of protein targets with more representatives that exhibit a higher conformational sampling. In order to minimize bias, an exhaustive inverse molecular docking was performed, and retrospective metric validation of our results indeed demonstrated enrichment.

**Figure 5:**
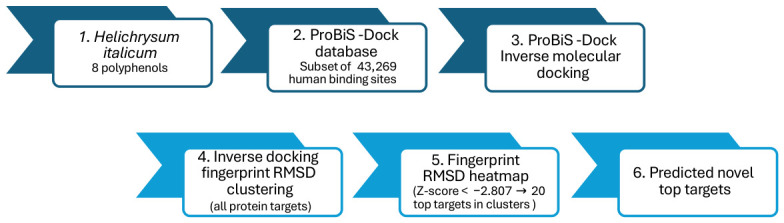
Workflow of the applied methodology. Eight *Helichrysum italicum* polyphenols were inversely docked into all human protein binding sites from the ProBiS-Dock database using ProBiS-Dock. Inverse molecular docking fingerprint clustering was applied to compare the polyphenol binding patterns. Top scoring targets were identified as proteins with low average Z-scores (below −2.807) with a cluster of polyphenols resulting from fingerprint clustering. Clustering was visually presented with the inverse molecular docking fingerprint RMSD heatmap of the top scoring targets, which were further discussed as potential protein targets of *Helichrysum italicum* polyphenols.

The average and standard deviation of the docking scores across all protein targets were calculated for each individual ligand, and the results were presented as ranked Z-scores, with a more negative Z-score denoting a stronger protein–ligand interaction. The protein target docking score distribution of each individual ligand closely follows a normal distribution (Supporting Information, Figures S19–S26). Detailed information about protein structures with PDB IDs and chains, as well as docking scores, is presented in Supporting Information. The applied methodology is schematically presented in Figure 5.

#### 3.1.1. Hierarchical Clustering and Inverse Molecular Docking Fingerprints

The inverse molecular docking methodology has been successfully applied and validated for identifying potential protein targets of various ligands [38,39,40]. In this study, a complementary inverse molecular docking fingerprint methodology [41] was additionally applied. The goal of fingerprinting is to compare the binding patterns of different compounds (polyphenols). To that end, the docking Z-scores between a given compound and protein targets were employed as a fingerprint for that compound. The fingerprint for each compound *l* is represented by the array of $N_{t}$ docking Z-scores, $Z_{l}$, where $Z_{l}\left(i\right)$ corresponds to the docking Z-score for compound *l* with the *i*-th protein target and $N_{t}$ is the total number of unique protein targets in the study. The RMSD matrix R, where each (l,k) ligand pair represents the RMSD between the Z-scores of these two ligands, was constructed according to Equation (1),

(1) $$R\left(l,k\right)=\mathrm{RMSD}\left(l,k\right)=\sqrt{\frac{1}{N_{t}}\sum_{i=1}^{N_{t}}{\left(Z_{l}\left(i\right)-Z_{k}\left(i\right)\right)}^{2}}\;.$$

Ligand binding patterns were analyzed using agglomerative hierarchical clustering with ward linkage (minimum variance approach) [135] of R and the results were presented as a dendrogram, in which clusters represent ligands with similar protein target binding patterns. The clusters of polyphenols with similar protein target binding patterns, obtained from the hierarchical fingerprint clustering, were used to study possible synergistic effects. The best-scoring targets were obtained with a newly introduced methodology, analyzing the average Z-scores of a given target protein across all polyphenols from a particular cluster. The protein targets with highly favorable average Z-scores (Z-score < −2.807, 99.75th percentile) within a polyphenol cluster were analyzed in detail. These top-scoring targets were applied to further demonstrate the trends in investigated *Helichrysum italicum* polyphenol binding patterns by plotting the Z-scores of individual polyphenols with the top-targets as a heatmap [41], visually demonstrating the fingerprinting methodology. Moreover, the top-scoring protein targets were inspected in detail, emphasizing the ones that exhibit favorable Z-scores as the most probable target proteins of polyphenols from the same fingerprint cluster. These protein targets represent the candidates with the largest potential for subsequent computational or experimental studies to confirm the therapeutic potential and possible synergistic effects of studied *Helichrysum italicum* polyphenols and their connection with diseases. Furthermore, the druggability of the top-scoring protein targets was investigated with the DoGSiteScorer web server [53], which is based on a machine-learning algorithm that determines the druggability score between 0 and 1 to a protein binding site, where a higher value denotes higher druggability. The introduced inverse molecular docking fingerprint methodology can be expanded to study binding patterns of various natural and synthetic compounds and to provide valuable insights into the binding patterns of structurally similar compounds, leading to the discovery of new molecular mechanisms and to the repurposing of existing drugs.

#### 3.1.2. Method Validation

Validation of the applied methodology was performed by applying established retrospective metrics, namely, receiver-operating characteristics (ROC) curves, robust initial enhancement (RIE), enrichment factors (EF1%), and Boltzmann-enhanced discrimination of ROC (BEDROC) [136]. The area under the ROC curve (AUC) yields the method’s predictive performance, where an AUC value above 0.5 indicates that the performance of the method is better than random. The early recognition of experimentally confirmed protein targets is quantified by RIE, EF1%, and BEDROC metrics. The experimentally validated protein targets of the eight investigated polyphenols were obtained from the ChEMBL database [137]. The validation was performed on flavonols kaempferol and quercetin as well as on flavanone naringenin, due to a sufficient amount of available experimental data on their biological activities in the ChEMBL database. The protein targets with a pChEMBL score higher than 4 were considered as experimentally confirmed targets [138]. pChEMBL score is defined as $-\log_{10}$(molar IC_50_, XC_50_, EC_50_, AC_50_, $K_{i}$, $K_{d}$, or Potency). The validation using these retrospective metrics ensures a robust performance and predictive power of our inverse docking fingerprint approach, allowing for the identification of potential protein targets of investigated polyphenols with relevant pharmacological activities.

**Figure 6:**
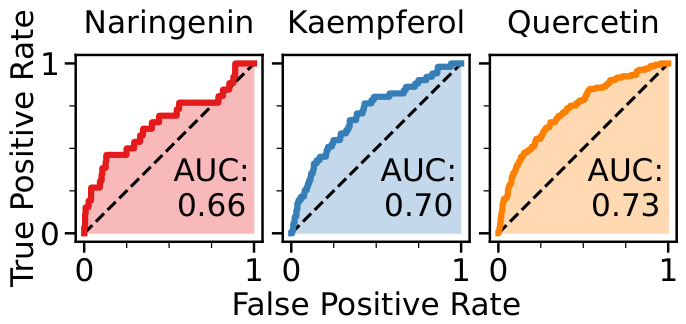
Receiver operating characteristics (ROC) curves for inverse molecular docking results of flavonols kaempferol and quercetin, as well as flavanone naringenin.

From Table 3 and Figure 6 it can be observed that the inverse molecular docking methodology produces valuable enrichment and can successfully identify experimentally confirmed protein targets.

Naringenin, kaempferol, and quercetin demonstrated favorable results, with ROC AUC of 0.66, 0.70, and 0.73, respectively. Moreover, the early target detection metrics were promising, with RIE of 4.31, 3.10, and 3.71, EF 1% of 15.09, 1.94, and 3.72, as well as BEDROC of 0.23, 0.18, and 0.26 for naringenin, kaempferol, and quercetin, respectively. The obtained retrospective metrics demonstrated a favorable predictive power (performance) of the inverse molecular docking protocol. However, the obtained early target detection metrics could be further enhanced in future studies by improving the database of docked structures. The applied ProBiS-Dock database [37] introduces some bias via unequal representation of protein structures, and further de-duplication would likely result in improved enrichment. Including organic cofactors, conserved water molecules, as well as the assignation of protein ionization states, all represent possible future methodological improvements.

## 4. Conclusions

In this study, the inverse molecular docking fingerprint approach based on hierarchical clustering was introduced to analyze the binding patterns of flavonol, flavanone and α-pyrone-class polyphenols from *Helichrysum italicum*, which resulted in three clusters, indicating similarities and differences in target binding patterns. The analysis of established fingerprints revealed that structurally similar flavonols and flavanones share similar binding patterns; however, unique binding affinity to specific protein targets can be observed for each polyphenol due to minor structural differences. These findings suggest that the mechanisms of action of each individual polyphenol should be examined in detail. From the top-scoring protein target list, specific binding patterns of polyphenolic compounds from flavonol, flavanone and α-pyrone classes can be identified together with their potential synergistic effects. Favorable docking scores of eight *Helichrysum italicum* polyphenols to two important oncogenic and antidiabetic targets, namely, CARM1 and PPARG, were identified for the first time, indicating synergistic effects. The unique top scoring protein targets of flavonol, flavanone and α-pyrone class polyphenols, related to cancer, neurodegeneration, and osteoporosis, were also identified, which should be further explored in computational and experimental studies. We firmly believe that this study also paves the way for future experimental in vitro and in vivo as well as computational validation of the predicted protein targets, likely leading to new therapeutic applications of *Helichrysum italicum* extracts and their polyphenolic compounds. Moreover, the inverse molecular docking fingerprint methodology possesses the potential to lead to the discovery of potential protein targets of various natural compounds, to the repurposing of existing drugs, and to the discovery and elucidation of molecular mechanisms or potential adverse effects.

## Figures and Tables

**Table 3:** Inverse molecular docking validation by retrospective metrics for selected polyphenols kaempferol, quercetin, and naringenin.

Ligand	AUC	EF 1%	RIE	BEDROC	$N_{\mathrm{actives}}$
Naringenin	0.66	15.09	4.31	0.23	26
Kaempferol	0.70	1.94	3.10	0.18	51
Quercetin	0.73	3.72	3.71	0.26	133

## Data Availability

The original contributions presented in this study are included in the article/Supplementary Material. Further inquiries can be directed to the corresponding authors.

## Supplementary Materials

The following supporting information can be downloaded at: https://www.mdpi.com/article/10.3390/ph19040647/s1.
