# Supplementary material for: Insights into Molecular Mechanisms of Polyphenolic Compounds from Helichrysum italicum by Inverse Molecular Docking Fingerprint Approach
Source: Pharmaceuticals (Basel). 2026 Apr 21;19(4):647. doi: 10.3390/ph19040647 (PMC13118441; doi:10.3390/ph19040647)
Supplement: Supplementary file 1 [file pharmaceuticals-19-00647-s001.zip › pharmaceuticals-4207618-supplementary.pdf]

Insights into Molecular Mechanisms of Polyphenolic Compounds from *Helichrysum italicum* by Inverse Molecular Docking Fingerprint Approach

Veronika Furlan, Vid Ravnik, Urban Bren*, and Marko Jukić*

*Email: urban.bren@um.si (U.B.), marko.jukic@um.si (M.J.)

Supplementary Materials

1 Results for fingerprint clustering

Figure S1 shows a heatmap plot of the all against all ligand docking Z-score RMSD matrix, $\mathcal{R}$, which results in the clustering dendrogram plot in the main text (Figure 3).

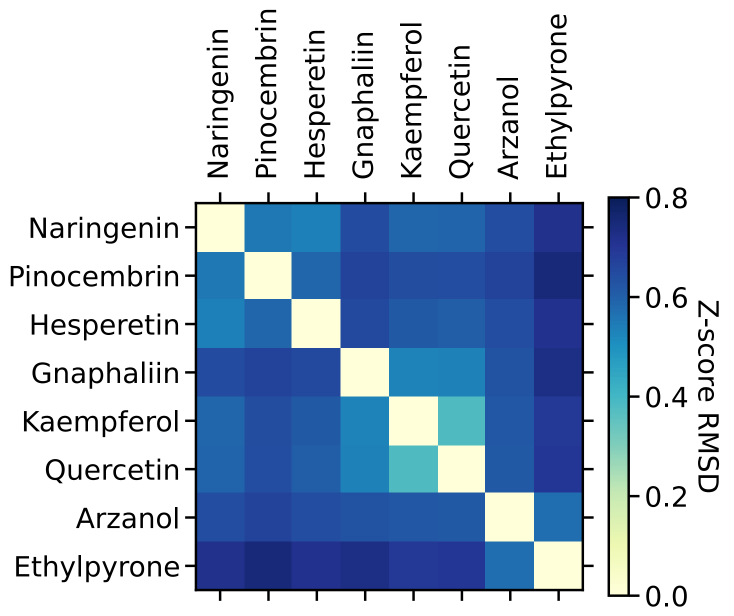

Figure S1: Heatmap plot of the all against all ligand docking Z-score RMSD matrix, $\mathcal{R}$. Results for fingerprints with all protein targets.

2 Results for fingerprint heatmap and potential novel targets

Table S1 shows numerical docking Z-score values for all polyphenols with all potential novel targets discussed in the main text. Docking attempts that failed are assigned a Z-score of +4.

Table S1: Individual Z score values for potential novel targets form main text.

Uniprot ID	Hesperetin	Pinocembrin	Naringenin	Quercetin	Kaempferol	Gnaphalin	Ethylpyrone	Arzanol
Q86X55	-2.49	-2.91	-3.07	-2.72	-2.17	-3.92	-3.24	-3.77

Continued on next page

Table S1: Individual Z score values for potential novel targets form main text.

Uniprot ID	Hesperetin	Pinocembrin	Naringenin	Quercetin	Kaempferol	Gnaphalin	Ethylpyrone	Arzanol
P37231	-2.84	-2.55	-3.53	-3.42	-2.73	-2.73	-2.93	-3.43
P00746	-2.24	-2.47	-3.25	-3.84	-3.35	-3.50	-2.65	-3.28
Q9NRG4	-3.29	-2.06	-2.80	-2.77	-2.58	-3.71	-2.84	-3.66
P14324	-1.75	-2.13	-2.12	-3.04	-3.03	-3.16	-3.29	-3.16
P00374	-3.72	-2.94	-3.02	-3.06	-2.86	-3.00	-2.09	-2.50
P11309	-2.87	-3.01	-3.10	-2.43	-3.02	-3.15	-2.06	-1.87
Q14832	-2.02	-1.56	-2.52	-3.38	-3.56	-2.84	-1.40	-2.70
Q9NPB1	-1.75	-1.85	-1.75	-3.40	-3.94	-4.18	-2.46	-2.97
O95372	-2.30	-2.65	-2.84	-3.07	-2.37	-3.50	-1.19	-1.32
O60760	-2.18	-3.13	-2.63	-3.07	-3.30	-4.03	-2.17	-2.13
Q53GL7	-1.45	-1.84	-2.53	-2.76	-3.32	-2.73	-1.13	-1.15
P01116	0.73	-0.23	-0.07	-2.51	-2.93	-1.32	-1.25	-1.13
P56817	-3.03	-2.36	-3.28	-2.19	-1.81	-2.68	-2.22	-2.76
Q92731	-3.25	-1.99	-3.29	-2.88	-2.33	-2.25	-0.89	-1.62
Q9H4B4	-1.70	-0.96	-1.50	-1.73	-1.64	-2.16	-3.00	-2.86
P24941	-2.47	-2.11	-2.80	-1.98	-1.99	-2.91	-2.87	-2.75
Q02750	-1.68	-1.75	-2.42	-2.70	-2.82	-2.39	-3.04	-2.81
Q14108	-1.41	-1.38	-1.09	-1.32	-1.34	-1.34	-2.78	-3.01
P09874	-1.59	-1.98	-2.17	-2.21	-2.02	-2.50	-3.18	-2.46

3 Detailed docking results for the most promising targets, PPARG and CARM1

In this section, we provide detailed results for the docking of all polyphenols to the two highest scoring targets, histone-arginine methyltransferase (CARM1) and peroxisome proliferator-activated receptor gamma (PPARG). Figure S2 shows the docking Z-scores for both targets.

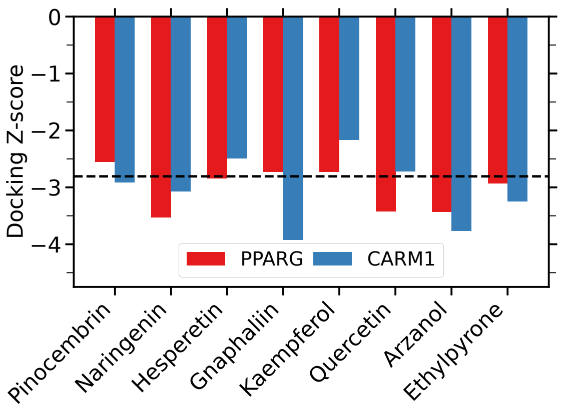

Figure S2: The bar chart displays the docking Z-scores of all studied polyphenols against the top-scoring targets, PPARG (red) and CARM1 (blue). The dashed line indicates the cutoff Z-score of −2.807 (99.75th percentile) applied throughout this study.

The binding interactions of the studied polyphenols with CARM1 and PPARG were further analyzed using the Protein-Ligand Interaction Profiler (PLIP). The interactions for each polyphenol are presented in Tables S4–S11, and the corresponding binding poses of polyphenols at the active site of CARM1 are visualized in Figures S3–S10. For PPARG, the individual PLIP interaction results are reported in Tables S12–S19 and Figures S11–S18.

The amino acid residues OF CARM1, involved in each type of interaction with polyphenols, were collated and are presented in Table S2. The GLU-257 residue of CARM1 was observed to play a major role in polyphenol binding, forming predominantly hydrophobic and hydrogen-bond interactions. Other prominent residues included TYR-153 (through hydrogen bonding, hydrophobic, and π-π interactions), HIS-414 (through hydrophobic and hydrogen-bonding interactions), and TYR-149 (through hydrogen bonding).

For PPARG, the collated PLIP results are presented in Table S3. ARG-288 was found to participate in key hydrophobic, hydrogen-bonding, and π-cation interactions, whereas ILE-341 frequently formed prominent hydrophobic interactions. GLU-343 was also identified as an important residue, forming hydrogen bonds and occasional hydrophobic contacts.

Table S2: Common interactions of studied polyphenols with CARM1 residues. Counts represent the number of a specific type of interaction formed by each residue across all polyphenols according to PLIP.

Residue	Interaction type count			
	H-bonds	Hydrophobic	π–cation	π–π
GLU-257	4	9	0	0
TYR-153	2	3	0	2
HIS-414	3	3	0	0
TYR-149	6	0	0	0
TRP-415	1	3	0	1
ARG-168	2	1	2	0
LEU-198	2	2	0	0
GLU-214	3	0	0	0
GLU-266	3	0	0	0
ILE-197	1	2	0	0

Table S3: Common interactions of studied polyphenols with PPARG residues. Counts represent the number of a specific type of interaction formed by each residue across all polyphenols according to PLIP.

Residue	Interaction type count			
	H-bonds	Hydrophobic	π–cation	π–π
ARG-288	5	5	1	0
ILE-341	0	9	0	0
GLU-343	5	2	0	0
SER-342	5	0	0	0
SER-332	4	0	0	0
LYS-263	3	0	0	0
TYR-222	3	0	0	0
PHE-287	0	2	0	1
ILE-262	1	2	0	0
ILE-326	1	1	0	0

3.1 Docking with CARM1

3.1.1 Pinocembrin

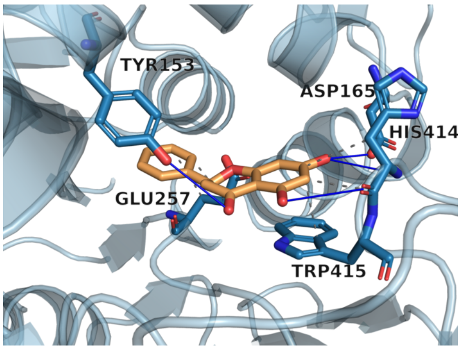

Figure S3: Docked pose and interactions of pinocembrin with CARM1

Table S4: Docked interactions of pinocembrin with CARM1 (6S7C-A) determined by PLIP

Residue	Interaction	Distance (Å)
GLU-257	Hydrophobic	3.97
GLU-257	Hydrophobic	3.69
HIS-414	Hydrophobic	3.50
HIS-414	Hydrophobic	3.86
TRP-415	Hydrophobic	3.62
TYR-153	H-bond	3.79
ASP-165	H-bond	2.75
HIS-414	H-bond	3.81
HIS-414	H-bond	3.75

3.1.2 Naringenin

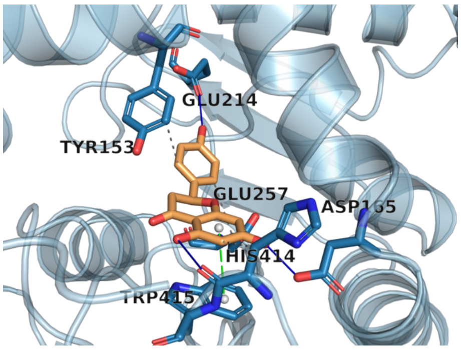

Figure S4: Docked pose and interactions of naringenin with CARM1

Table S5: Docked interactions of naringenin with CARM1 (6S79-A) determined by PLIP

Residue	Interaction	Distance (Å)
TYR-153	Hydrophobic	3.63
GLU-257	Hydrophobic	3.97
HIS-414	Hydrophobic	3.61
ASP-165	H-bond	2.98
GLU-214	H-bond	3.34
HIS-414	H-bond	3.98
TRP-415	π–π	3.99

3.1.3 Hesperetin

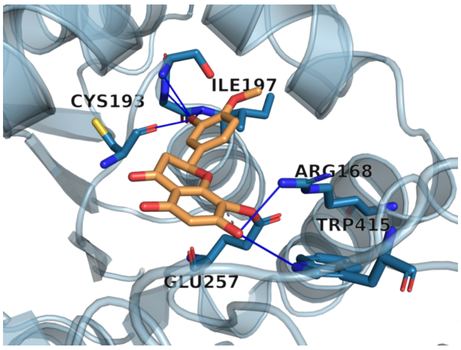

Figure S5: Docked pose and interactions of hesperetin with CARM1

Table S6: Docked interactions of hesperetin with CARM1 (6S7A-C) determined by PLIP

Residue	Interaction	Distance (Å)
GLU-257	Hydrophobic	3.71
ARG-168	H-bond	3.51
CYS-193	H-bond	2.19
SER-195	H-bond	3.12
GLY-196	H-bond	3.03
ILE-197	H-bond	3.15
TRP-415	H-bond	3.38

3.1.4 Gnaphalin

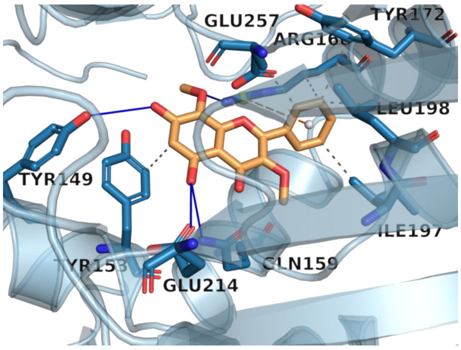

Figure S6: Docked pose and interactions of gnaphalin with CARM1

Table S7: Docked interactions of gnaphalin with CARM1 (6S7A-C) determined by PLIP

Residue	Interaction	Distance (Å)
TYR-153	Hydrophobic	3.91
ARG-168	Hydrophobic	3.91
TYR-172	Hydrophobic	3.75
ILE-197	Hydrophobic	3.75
LEU-198	Hydrophobic	3.77
GLU-257	Hydrophobic	3.22
TYR-149	H-bond	3.94
GLN-159	H-bond	3.94
ARG-168	H-bond	3.73
GLU-214	H-bond	3.76
ARG-168	π–cation	5.44

3.1.5 Kaempferol

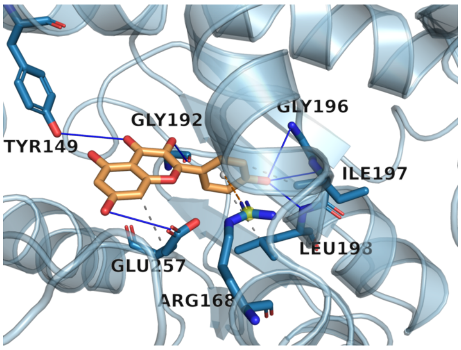

Figure S7: Docked pose and interactions of kaempferol with CARM1

Table S8: Docked interactions of kaempferol with CARM1 (6S7C-A) determined by PLIP

Residue	Interaction	Distance (Å)
ILE-197	Hydrophobic	3.43
LEU-198	Hydrophobic	3.68
GLU-257	Hydrophobic	3.62
TYR-149	H-bond	4.09
GLY-192	H-bond	2.41
GLY-196	H-bond	3.83
GLY-196	H-bond	4.08
LEU-198	H-bond	2.89
GLU-257	H-bond	3.59
GLU-257	H-bond	3.59
ARG-168	π–cation	5.00

3.1.6 Quercetin

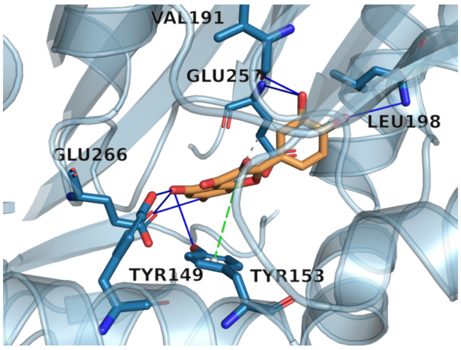

Figure S8: Docked pose and interactions of quercetin with CARM1

Table S9: Docked interactions of quercetin with CARM1 (6S7B-C) determined by PLIP

Residue	Interaction	Distance (Å)
GLU-257	Hydrophobic	3.94
GLU-257	Hydrophobic	3.96
TYR-149	H-bond	2.84
TYR-153	H-bond	3.60
VAL-191	H-bond	2.94
LEU-198	H-bond	3.77
GLU-257	H-bond	3.80
GLU-266	H-bond	3.81
GLU-266	H-bond	2.76
TYR-153	π–π	5.06

3.1.7 Arzanol

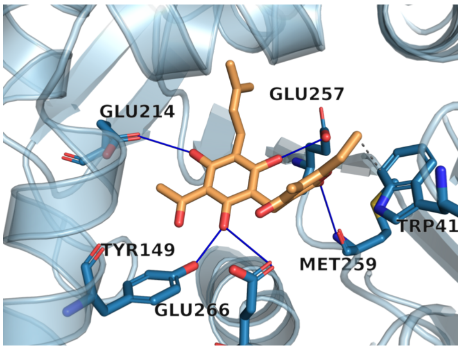

Figure S9: Docked pose and interactions of arzanol with CARM1

Table S10: Docked interactions of arzanol with CARM1 (6S7B-C) determined by PLIP

Residue	Interaction	Distance (Å)
TRP-415	Hydrophobic	3.74
TYR-149	H-bond	2.73
GLU-214	H-bond	3.64
GLU-257	H-bond	2.95
MET-259	H-bond	4.09
GLU-266	H-bond	3.67

3.1.8 Ethylpyrone

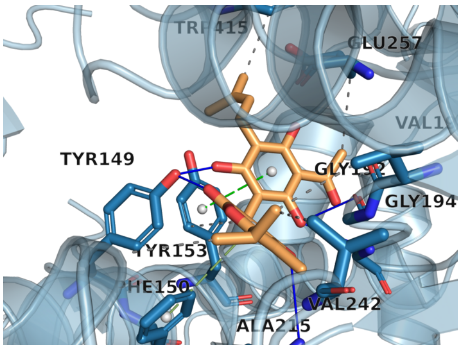

Figure S10: Docked pose and interactions of ethylpyrone with CARM1

Table S11: Docked interactions of ethylpyrone with CARM1 (6S74-A) determined by PLIP

Residue	Interaction	Distance (Å)
PHE-137	Hydrophobic	3.81
TYR-153	Hydrophobic	3.84
VAL-191	Hydrophobic	3.72
VAL-242	Hydrophobic	3.15
GLU-257	Hydrophobic	4.00
TRP-415	Hydrophobic	3.63
TYR-149	H-bond	2.04
TYR-149	H-bond	2.21
GLY-192	H-bond	3.05
GLY-194	H-bond	3.58
ALA-215	H-bond	3.94
PHE-150	π–π	5.35
TYR-153	π–π	4.45

3.2 Docking with PPARG

3.2.1 Pinocembrin

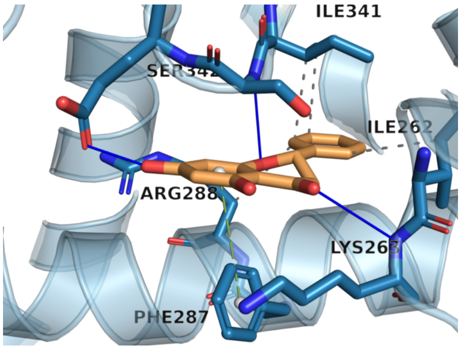

Figure S11: Docked pose and interactions of pinocembrin with PPARG

Table S12: Docked interactions of pinocembrin with PPARG (6D8X-A) determined by PLIP

Residue	Interaction	Distance (Å)
ILE-262	Hydrophobic	3.83
ARG-288	Hydrophobic	3.85
ILE-341	Hydrophobic	3.87
ILE-341	Hydrophobic	3.72
LYS-263	H-bond	3.99
SER-342	H-bond	3.51
GLU-343	H-bond	2.49
GLU-343	H-bond	2.49
PHE-287	π–π	5.16

3.2.2 Naringenin

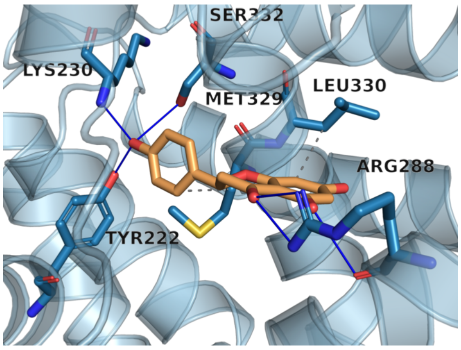

Figure S12: Docked pose and interactions of naringenin with PPARG

Table S13: Docked interactions of naringenin with PPARG (2FVJ-A) determined by PLIP

Residue	Interaction	Distance (Å)
MET-329	Hydrophobic	3.60
LEU-330	Hydrophobic	3.82
TYR-222	H-bond	2.48
LYS-230	H-bond	2.61
ARG-288	H-bond	3.48
ARG-288	H-bond	3.02
ARG-288	H-bond	3.09
SER-332	H-bond	3.60
SER-332	H-bond	3.60

3.2.3 Hesperetin

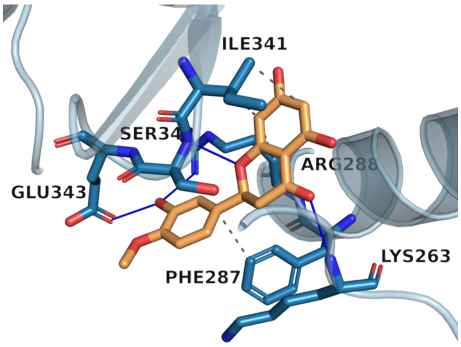

Figure S13: Docked pose and interactions of hesperetin with PPARG

Table S14: Docked interactions of hesperetin with PPARG (6D8X-A) determined by PLIP

Residue	Interaction	Distance (Å)
PHE-287	Hydrophobic	3.62
ARG-288	Hydrophobic	3.95
ILE-341	Hydrophobic	3.49
ILE-341	Hydrophobic	3.30
LYS-263	H-bond	3.08
ARG-288	H-bond	3.95
SER-342	H-bond	3.10
GLU-343	H-bond	2.56
GLU-343	H-bond	2.56

3.2.4 Gnaphalin

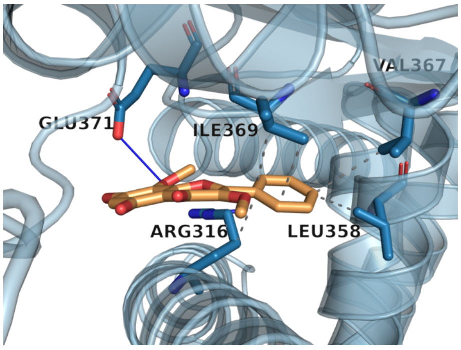

Figure S14: Docked pose and interactions of gnaphalin with PPARG

Table S15: Docked interactions of gnaphalin with PPARG (6FZG-A) determined by PLIP

Residue	Interaction	Distance (Å)
ARG-316	Hydrophobic	3.68
LEU-358	Hydrophobic	3.37
VAL-367	Hydrophobic	3.78
ILE-369	Hydrophobic	3.50
ILE-369	Hydrophobic	3.37
GLU-371	H-bond	3.21

3.2.5 Kaempferol

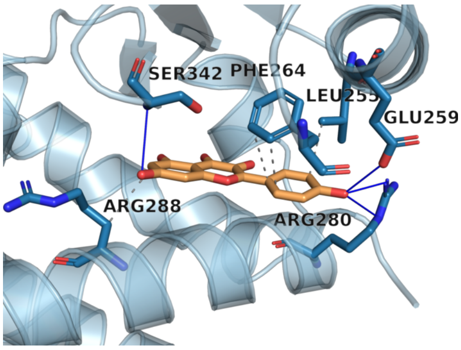

Figure S15: Docked pose and interactions of kaempferol with PPARG

Table S16: Docked interactions of kaempferol with PPARG (6DGL-A) determined by PLIP

Residue	Interaction	Distance (Å)
LEU-255	Hydrophobic	3.88
PHE-264	Hydrophobic	3.44
PHE-264	Hydrophobic	3.66
ARG-288	Hydrophobic	3.96
GLU-259	H-bond	2.96
ARG-280	H-bond	2.88
ARG-280	H-bond	3.59
SER-342	H-bond	3.65

3.2.6 Quercetin

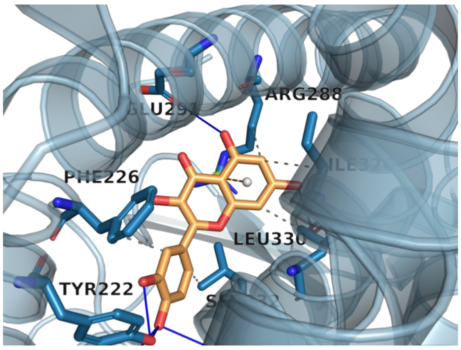

Figure S16: Docked pose and interactions of quercetin with PPARG

Table S17: Docked interactions of quercetin with PPARG (6O68-A) determined by PLIP

Residue	Interaction	Distance (Å)
PHE-226	Hydrophobic	3.79
ARG-288	Hydrophobic	3.11
ILE-326	Hydrophobic	3.90
LEU-330	Hydrophobic	3.48
LEU-333	Hydrophobic	3.42
TYR-222	H-bond	2.88
TYR-222	H-bond	2.43
LYS-230	H-bond	2.86
GLU-291	H-bond	4.01
GLU-291	H-bond	4.01
ILE-326	H-bond	3.84
SER-332	H-bond	3.87
SER-332	H-bond	3.87
ARG-288	π–cation	4.07

3.2.7 Arzanol

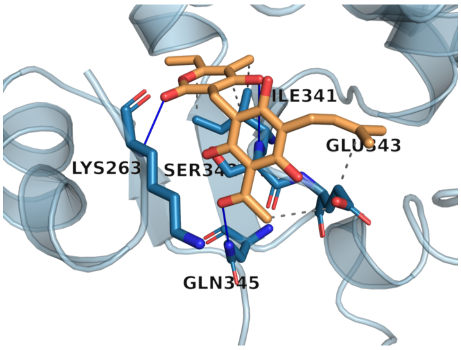

Figure S17: Docked pose and interactions of arzanol with PPARG

Table S18: Docked interactions of arzanol with PPARG (5GTP-A) determined by PLIP

Residue	Interaction	Distance (Å)
ILE-341	Hydrophobic	3.84
ILE-341	Hydrophobic	3.55
ILE-341	Hydrophobic	3.41
GLU-343	Hydrophobic	3.94
GLU-343	Hydrophobic	3.50
LYS-263	H-bond	3.19
SER-342	H-bond	4.07
GLN-345	H-bond	2.61

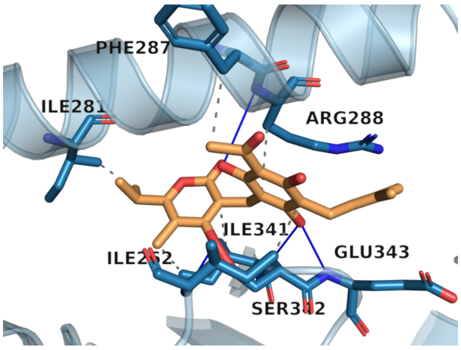

Figure S18: Docked pose and interactions of ethylpyrone with PPARG

Table S19: Docked interactions of ethylpyrone with PPARG (3U9Q-A) determined by PLIP

Residue	Interaction	Distance (Å)
ILE-262	Hydrophobic	3.37
ILE-281	Hydrophobic	2.82
PHE-287	Hydrophobic	3.56
ARG-288	Hydrophobic	3.88
ILE-341	Hydrophobic	3.57
ILE-341	Hydrophobic	3.86
ILE-262	H-bond	3.51
ARG-288	H-bond	4.06
SER-342	H-bond	2.70
GLU-343	H-bond	2.89

3.2.8 Ethylpyrone

4 Results for individual polyphenols

The following sections include histograms displaying the distribution of docking scores for each polyphenol (blue), accompanied by a plot of a normal distribution with corresponding mean and standard deviation (orange). The dashed red lines represent Z-scores below -2.81. Tables of the top 10 highest-scoring protein targets for each investigated *Helichrysum italicum* polyphenol are also provided.

4.1 Naringenin

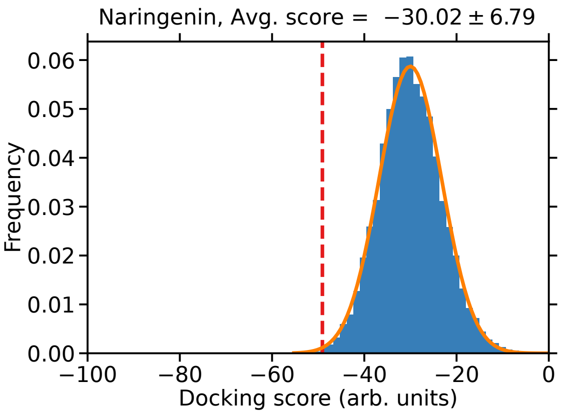

Figure S19: Histogram of docking scores for naringenin

Table S20: Top 10 protein targets for naringenin

UniProt ID	PDB ID - Chain	Protein Name	Docking Score (arb. units)	Z-Score
P37231	3U9Q-A	Peroxisome proliferator-activated receptor gamma	-53.98	-3.53
Q92731	1QKM-A	Estrogen receptor beta	-52.34	-3.29
P56817	5QD9-A	Beta-secretase 1	-52.30	-3.28
P00746	5FBE-A	Complement factor D	-52.10	-3.25
P16442	4FRE-A	Histo-blood group ABO system transferase	-51.84	-3.21
P09237	2MZI-A	Matrilysin	-51.31	-3.13
P11309	5KGK-A	Serine/threonine-protein kinase pim-1	-51.08	-3.10
O75469	4NY9-A	Nuclear receptor subfamily 1 group I member 2	-51.01	-3.09
Q86X55	6S74-A	Histone-arginine methyltransferase CARM1	-50.86	-3.07
P00374	1KMS-A	Dihydrofolate reductase	-50.54	-3.02

4.2 Pinocembrin

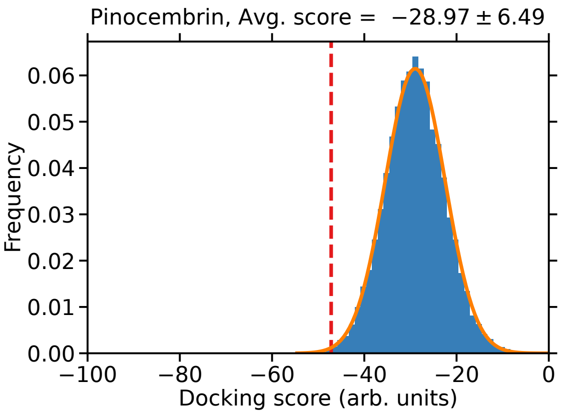

Figure S20: Histogram of docking scores for pinocembrin

Table S21: Top 10 protein targets for pinocembrin

UniProt ID	PDB ID - Chain	Protein Name	Docking Score (arb. units)	Z-Score
P14061	1BHS-A	17-beta-hydroxysteroid dehydrogenase type 1	-49.37	-3.14
O60760	6N4E-A	Hematopoietic prostaglandin D synthase	-49.26	-3.13
P40261	6B1A-A	Nicotinamide N-methyltransferase	-49.05	-3.09
O75469	4NY9-A	Nuclear receptor subfamily 1 group I member 2	-48.51	-3.01
P11309	5KGK-A	Serine/threonine-protein kinase pim-1	-48.48	-3.01
P00374	1KMS-A	Dihydrofolate reductase	-48.06	-2.94
Q92831	6J3O-A	Histone acetyltransferase KAT2B	-48.03	-2.94
Q460N5	6FYM-A	Protein mono-ADP-ribosyltransferase PARP14	-47.91	-2.92
Q86X55	6S74-A	Histone-arginine methyltransferase CARM1	-47.84	-2.91
O14744	6K1S-A	Protein arginine N-methyltransferase 5	-47.79	-2.90

4.3 Gnaphaliin

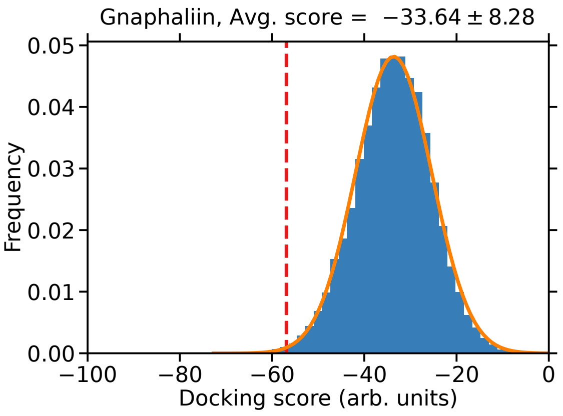

Figure S21: Histogram of docking scores for gnaphaliin

Table S22: Top 10 protein targets for gnaphaliin

UniProt ID	PDB ID - Chain	Protein Name	Docking Score (arb. units)	Z-Score
Q9NPB1	1Z4J-A	5'(3')-deoxyribonucleotidase, mitochondrial	-68.26	-4.18
O60760	6N4E-A	Hematopoietic prostaglandin D synthase	-67.04	-4.03
Q86X55	6S74-A	Histone-arginine methyltransferase CARM1	-66.12	-3.92
Q96PN6	4OZ2-A	Adenylate cyclase type 10	-65.41	-3.84
Q9NRG4	4O6F-A	N-lysine methyltransferase SMYD2	-64.35	-3.71
O95372	5SYN-A	Acyl-protein thioesterase 2	-62.61	-3.50
P00746	5FBE-A	Complement factor D	-62.58	-3.50
O95749	6G31-F	Geranylgeranyl pyrophosphate synthase	-59.98	-3.18
P08631	5H0G-A	Tyrosine-protein kinase HCK	-59.84	-3.17
P14324	1YV5-A	Farnesyl pyrophosphate synthase	-59.83	-3.16

4.4 Kaempferol

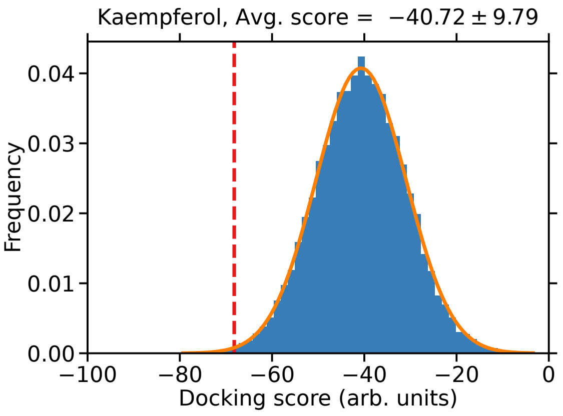

Figure S22: Histogram of docking scores for kaempferol

Table S23: Top 10 protein targets for kaempferol

UniProt ID	PDB ID - Chain	Protein Name	Docking Score (arb. units)	Z-Score
Q9NPB1	1Z4J-A	5'(3')-deoxyribonucleotidase, mitochondrial	-79.34	-3.94
Q14832	6B7H-A	Metabotropic glutamate receptor 3	-75.60	-3.56
P00746	5FBE-A	Complement factor D	-73.51	-3.35
Q53GL7	6FXI-A	Protein mono-ADP-ribosyltransferase PARP10	-73.22	-3.32
O60760	6N4E-A	Hematopoietic prostaglandin D synthase	-73.07	-3.30
Q460N5	6FYM-A	Protein mono-ADP-ribosyltransferase PARP14	-72.67	-3.26
Q16539	6QYX-A	Mitogen-activated protein kinase 14	-45.72	-0.51
P07814	5VAD-A	Bifunctional glutamate/proline–tRNA ligase	-71.42	-3.14
Q9H2K2	4BJ9-A	Poly [ADP-ribose] polymerase tankyrase-2	-70.71	-3.06
P14324	1YV5-A	Farnesyl pyrophosphate synthase	-70.40	-3.03

4.5 Arzanol

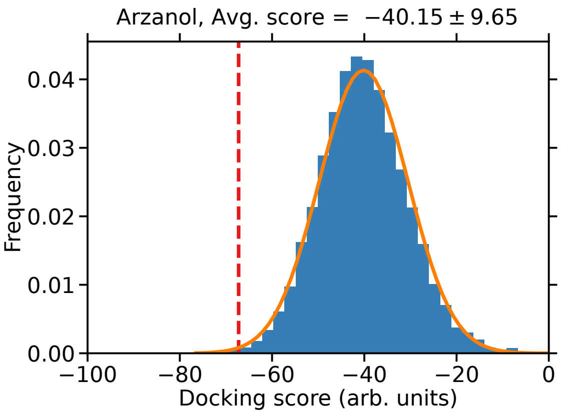

Figure S23: Histogram of docking scores for arzanol

Table S24: Top 10 protein targets for arzanol

UniProt ID	PDB ID - Chain	Protein Name	Docking Score (arb. units)	Z-Score
Q86X55	6S74-A	Histone-arginine methyltransferase CARM1	-76.51	-3.77
Q9NRG4	4O6F-A	N-lysine methyltransferase SMYD2	-75.52	-3.66
P37231	3U9Q-A	Peroxisome proliferator-activated receptor gamma	-73.29	-3.43
P00746	5FBE-A	Complement factor D	-71.77	-3.28
P14324	1YV5-A	Farnesyl pyrophosphate synthase	-70.62	-3.16
Q14108	4TW0-A	Lysosome membrane protein 2	-69.21	-3.01
Q9NPB1	1Z4J-A	5'(3')-deoxyribonucleotidase, mitochondrial	-68.78	-2.97
P05543	4X30-A	Thyroxine-binding globulin	-68.75	-2.96
Q9H4B4	4B6L-A	Serine/threonine-protein kinase PLK3	-67.73	-2.86
O75608	6QGO-B	Acyl-protein thioesterase 1	-67.39	-2.82

4.6 Quercetin

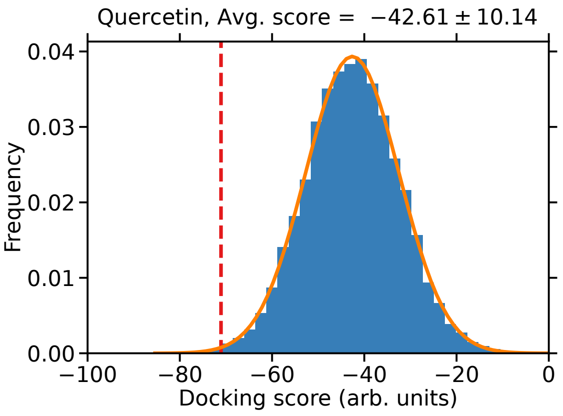

Figure S24: Histogram of docking scores for quercetin

Table S25: Top 10 protein targets for quercetin

UniProt ID	PDB ID - Chain	Protein Name	Docking Score (arb. units)	Z-Score
Q460N5	6FYM-A	Protein mono-ADP-ribosyltransferase PARP14	-83.88	-4.07
P00746	5FBE-A	Complement factor D	-81.52	-3.84
P37231	3U9Q-A	Peroxisome proliferator-activated receptor gamma	-77.30	-3.42
Q9NPB1	1Z4J-A	5'(3')-deoxyribonucleotidase, mitochondrial	-77.13	-3.40
Q14832	6B7H-A	Metabotropic glutamate receptor 3	-76.87	-3.38
P23919	1E9F-A	Thymidylate kinase	-69.15	-2.62
Q9UKK9	5QTM-B	ADP-sugar pyrophosphatase	-74.40	-3.14
O95372	5SYN-A	Acyl-protein thioesterase 2	-73.76	-3.07
O60760	6N4E-A	Hematopoietic prostaglandin D synthase	-73.71	-3.07
P00374	1KMS-A	Dihydrofolate reductase	-73.60	-3.06

4.7 Hesperetin

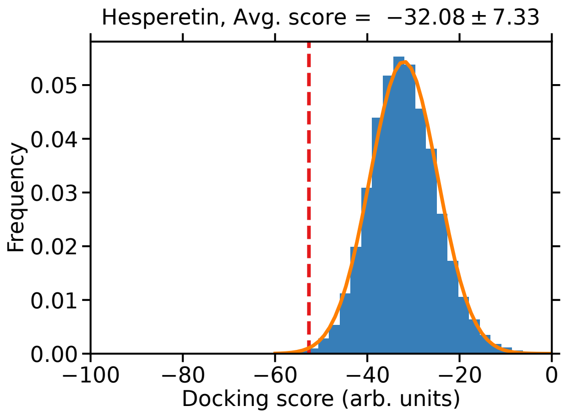

Figure S25: Histogram of docking scores for hesperetin

Table S26: Top 10 protein targets for hesperetin

UniProt ID	PDB ID - Chain	Protein Name	Docking Score (arb. units)	Z-Score
P00374	1KMS-A	Dihydrofolate reductase	-59.38	-3.72
P07814	5VAD-A	Bifunctional glutamate/proline–tRNA ligase	-56.76	-3.37
Q9NRG4	4O6F-A	N-lysine methyltransferase SMYD2	-56.18	-3.29
Q92731	1QKM-A	Estrogen receptor beta	-55.90	-3.25
Q460N5	6FYM-A	Protein mono-ADP-ribosyltransferase PARP14	-55.72	-3.22
P47989	2E1Q-A	Xanthine dehydrogenase/oxidase	-54.88	-3.11
P56817	5QD9-A	Beta-secretase 1	-54.31	-3.03
P55055	1P8D-A	Oxysterols receptor LXR-beta	-54.27	-3.03
P19367	4FPA-B	Hexokinase-1	-53.42	-2.91
P00751	2OK5-A	Complement factor B	-53.28	-2.89

4.8 Ethylpyrone

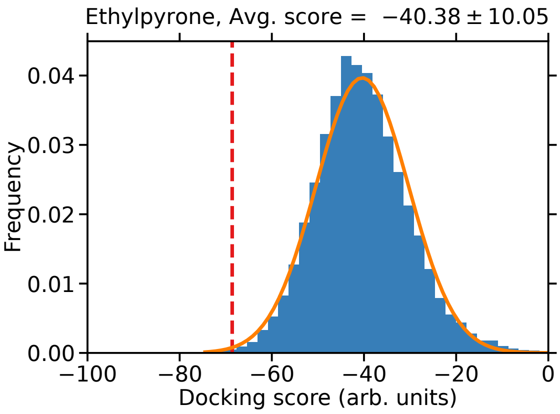

Figure S26: Histogram of docking scores for ethylpyrone

Table S27: Top 10 protein targets for ethylpyrone

UniProt ID	PDB ID - Chain	Protein Name	Docking Score (arb. units)	Z-Score
P14324	1YV5-A	Farnesyl pyrophosphate synthase	-73.50	-3.29
Q86X55	6S74-A	Histone-arginine methyltransferase CARM1	-72.99	-3.24
P09874	6VKK-A	Poly [ADP-ribose] polymerase 1	-72.37	-3.18
P00742	2XC0-A	Coagulation factor X	-71.34	-3.08
Q02750	3ORN-A	Dual specificity mitogen-activated protein kinase kinase 1	-70.91	-3.04
P11362	3KRL-A	Fibroblast growth factor receptor 1	-54.04	-1.36
Q9H4B4	4B6L-A	Serine/threonine-protein kinase PLK3	-70.59	-3.00
P37231	3U9Q-A	Peroxisome proliferator-activated receptor gamma	-69.88	-2.93
P24941	3R9H-A	Cyclin-dependent kinase 2	-69.20	-2.87
Q9UM73	4JOA-A	ALK tyrosine kinase receptor	-68.93	-2.84
